# Acute Type A aortic dissection surgical repair in Octogenarians: A meta-analysis

**DOI:** 10.1038/s44325-024-00007-9

**Published:** 2024-08-31

**Authors:** Mohammed Tarek Hasan, Hazem Salah Rezq, Othman Saleh, Heba Aboeldahab, Mohammad K. El khashab, Salah Mahmoud Hamouda, Hassan Elkersh, Mohamed Ibrahim Gbreel, Aly Sherif Hassaballa, Ahmed K. Awad

**Affiliations:** 1 Faculty of Medicine, Al-Azhar University, Cairo, Egypt; 2 Faculty of Medicine, The Hashemite University, Zarqa, Jordan; 3 Biomedical Informatics and Medical Statistics Department, Medical Research Institute, Alexandria University, Cairo, Egypt; 4 Faculty of Medicine, October 6 University, Giza, Egypt; 5 Department of Cardiothoracic Surgery, Faculty of Medicine, Ain Shams University, Cairo, Egypt; 6 Department of Cardiothoracic Surgery, Ain-Shams University hospitals, Cairo, Egypt; 7 Faculty of Medicine, Ain-shams University, Cairo, Egypt

**Keywords:** Cardiology, Interventional cardiology

## Abstract

Aortic dissection (AD), a life-threatening condition resulting from aortic wall tears, is especially concerning in the elderly. However, few studies have investigated long-term surgical outcomes in octogenarians with Type A aortic dissection (TAAD). Our paper addresses this critical knowledge gap. Four electronic databases were searched from inception till November 2022 to include any observational or randomized controlled trials (RCT) that evaluate long-term surgical outcomes of TAAD in octogenarians alone or compared with Septuagint focusing on factors including surgical approach, comorbidities, and preoperative status. The Mantel-Haenszel method was used to pool study estimates and calculate odds ratios (OR) with 95% confidence intervals (CI). We included 18,057 participants (10,253 males, 7804 females). In octogenarians and compared to medical treatment, surgical repair achieved significantly lower rates of re-exploration (9%), antegrade cerebral perfusion (33%), stroke (10%), and respiratory failure (19%). In terms of operative data, octogenarians had shorter cardiopulmonary bypass time (161.89 min), cross-clamp time (103.18 min), and myocardial ischemic time (90.89 min). Compared to septuagenarians, octogenarians had significantly shorter cardiopulmonary bypass and systemic cardiac arrest times (−13.84 min and −2.46 min, respectively). Additionally, octogenarians had a higher risk of respiratory complications (RR = 1.60). No significant differences were found for tracheostomy, antegrade cerebral perfusion, neurologic complications, and renal failure. In conclusion, octogenarians undergoing surgical repair for TAAD face relatively lower complication rates, but a higher risk of respiratory issues compared to septuagenarians, emphasizing the unique surgical challenges in this elderly fragile population.

## Introduction

Aortic dissection is a life-threatening medical condition that results from a disruption in the aortic wall integrity, specifically a tear in the intimal layer^1^. This condition leads to the formation of a false lumen within the aortic wall, allowing blood to flow between the intimal and medial layers causing the aorta to bulge or even rupture, resulting in severe complications as cardiac tamponade, stroke, or sudden death^1,2^.

A Stanford Type A aortic dissection (TAAD) refers to a specific classification system used to categorize aortic dissections based on the location of the tear in the aorta. In TAAD, the tear occurs in the ascending aorta, which is the portion of the aorta that rises from the heart. This type of dissection involves the ascending aorta and can extend further down the aorta as well^2^. Stanford Type A aortic dissections are considered surgical emergencies due to the risk of life-threatening complications such as aortic rupture, cardiac tamponade, or compromise of blood flow to vital organs. Immediate surgical intervention is typically required to repair the damaged aorta and prevent further complications^2^.

With the aging of the population, it is crucial to better understand the long-term outcomes associated with surgical intervention in individuals with TAAD who are at a higher risk^3^. Recent studies^4–7^ have shown that advanced age significantly predicts adverse outcomes following surgical treatment for TAAD. This is a concerning finding given the increasing prevalence of TAAD among individuals over 60 years, as well as the rising life expectancy of the general population^3^. As such, efforts to optimize surgical approaches and minimize the risk of complications are critical for improving outcomes in this patient population^5–7^.

Surgical intervention is almost always the standard of management for all cases of Stanford TAAD^8^. As mentioned before: surgery is the standard care in all cases of TAAD; however, the optimal surgical approach for octogenarians remains a topic of debate^9^. Furthermore, Long-term outcomes after surgical treatment for TAAD in octogenarians can be influenced not only by the surgical approach but also by other factors such as preoperative health status and comorbidities which increase unfavorable outcomes.

Therefore, we conducted the current systematic review and meta-analysis of published studies to assess the long-term surgical outcomes of TAAD in octogenarians, with a focus on factors including surgical approach, comorbidities, and preoperative status.

## Methods

We conducted the present study based on the Cochrane Handbook of Systematic Reviews on Interventions^10^. During the process of drafting our manuscript, we strictly followed the recommended reporting items for the Preferred Reporting Items for Systematic Reviews and Meta-Analyses (PRISMA) statement guidelines^11^ as well as the meta-analyses Of Observational Studies in Epidemiology (MOOSE)^12^. Also, the results were reported in line with AMSTAR-2 (Assessing the methodological quality of systematic reviews 2) guidelines^13^.

### Search strategy

We searched PubMed, Cochrane Library, Scopus, and Web of Science (WOS) for studies that investigate studies that performed a surgical repair for TAAD in octogenarians’ population to September 20, 2022. The following search terms were used: (type A aortic dissection OR type A OR aortic dissection OR TAAD repair OR dissection surgical repair) AND (octogenarians OR older than 80). Additionally, we reviewed the reference lists of eligible articles to complement the broad search.

### Eligibility criteria

The PICOS (population, intervention, comparator, outcome, study design) criteria of our study included: (P); patients aged equal to or greater than 80 years old or octogenarians and suffering from TAAD; (I) aortic surgical repair by any technique; (C) younger patients or Septuagint; (O) bypass time, cardiac arrest time, myocardial ischemia time, risk of neurological complications, re-exploration, renal failure, respiratory failure, stroke, and tracheostomy; (S) randomized controlled trials (RCTs) and Cohort studies. We excluded any other studies that did not follow our PICOS criteria including different study designs including conference papers, unpublished articles, letters to the editor, posters, and in vitro studies, non-English studies, studies that included patients with congenital heart diseases, or heart transplants.

### Data extraction

We extracted the following data from the included studies as baseline characteristics: study ID, publication year, country, study design, gender, mean age, total sample size, length of follow-up, name of surgical procedure, clinical background including hypertension, diabetes, atrial fibrillation, Smoking, COPD, Hyperlipidemia, Renal insufficiency, Coronary artery disease, Aortic valve regurgitation >2 degree. Furthermore, for qualitative and quantitative analysis, we extracted bypass time, cardiac arrest time, myocardial ischemia time, risk of neurological complications, re-exploration, renal failure, respiratory failure, stroke, and tracheostomy.

### Quality assessment

The risk of bias was assessed using the Cochrane tool the risk of bias-2 (ROB-2)^14^ according to the Cochrane handbook to assess RCTs and NIH quality assessment tool for observational studies^15^ to assess observational studies. Two independent reviewers (H.S.R and H.A.) screened the methodological quality of included studies and in case of discrepancies were resolved by senior author.

### Statistical analysis

The Mantel-Haenszel (M-H) method was used to pool study estimates, and the restricted maximum-likelihood estimator was used to estimate between-study heterogeneity. The risk ratio (RR) with a 95% confidence interval (CI) are provided as effect size estimates. Both fixed and random-effects meta-analysis models were utilized to investigate whether the results were sensitive to model choice. Forest plots were drawn, and the shaded boxes represent the point estimate for each individual trial, and the horizontal line extending from each box represents the upper and lower limits of the 95% CI. The diamonds represent the overall effect size.

Heterogeneity was evaluated using the Cochrane Q test, Chi-square test, and I2 statistic. I2 > 50% denoted substantial heterogeneity in the studies. A meta-analysis was performed using the random-effects model and inverse variance method. Funnel plots were used for visual investigation of publication bias and further assessed by Eggers statistics^16^. All analyses were conducted using R software version 4.0^17^. A *p*-value less than 0.05 was considered statistically significant.

## Results

### Literature search and study selection

Our search strategy resulted in a total number of 13426 studies. After the title and abstract screening and removing the duplicates, 38 full-text articles were evaluated for eligibility. Following the full-text screening, 27^18–44^ observational studies met our criteria and were included in our meta-analysis (Supplementary Fig. 1 and Supplementary Table 1).

### Characteristics of the included studies

Twenty-seven cohort studies with 18057 participants were included with a total 10,253 males and 7804 females. The institutional surgical databases that are used are: Fourteen studies were from Japan, three in Germany, three in the USA, two in France, one in England, one in Taiwan, and three studies from other databases. Among the included studies, all of them were retrospective except two studies were prospective. Detailed summary and baseline characteristics of the included studies are illustrated in (Table 1 and Supplementary Table 2).

**Table 1:** Summary of included studies

Study ID	Study site	Design	Total sample size	Length of follow-up	Name of the procedures
Bojko 2022	USA	Retrospective	235	1.4:3.7 years	Total arch replacement, Bentall-Aortic root replacement
Nakai 2022	Japan	Retrospective	188	0.25:10.6 years	Total arch replacement
Dumfarth 2017	Austria-Germany-USA	Retrospective	67	1.6 ± 2.7 years	Isolated ascending replacement, Root replacement, Aortic valve replacement, Aortic arch replacement, Coronary artery bypass grafting (CABG)
Hata 2008	Japan	Retrospective	269	No follow up	Ascending aorta replacement, Hemi arch replacement, Total arch replacement
Hata2010	Japan	Retrospective	27	2.2 years	Ascending aorta replacement, Hemi arch replacement
Kondoh 2016	Japan	Retrospective	436	No follow up	Ascending aorta replacement, Total arch replacement, Aortic root replacement, CABG
Ahmed 2015	Germany	Prospective	39	3.6 (2.8) years	Hemiarch replacement, Full arch replacement, Bio-Bentall.
Neri 2001	France & Italy	Retrospective	197		Ascending aortic replacement, Hemiarch replacement, Total arch replacement, Proximal procedure, Aorto-aortic graft, Bentall procedure, Cabrol procedure, Aortic valve resuspension
Piccardo 2009	France & Italy	Retrospective	57	3.9 (2) years	Ascending aortic replacement, Hemiarch replacement, Total arch replacement, Root aortic replacement
Shiono 2006	Japan	Retrospective	134	10 years	Ascending aortic replacement, Hemiarch replacement total arch replacement, Root replacement, Aortic valve valvuloplasty / Replacement.
Tang 2013	USA	Retrospective	101	1.4 (1.3) years	Ascending aortic replacement, Hemiarch replacement, Total arch replacement, Bentall procedure, David procedure, Aortic valve, and ascending aorta replacement (Wheat procedure)
Vanhuyse 2012	France	Retrospective	15	follow-up rate was 100% complete up to January 2011.	Ascending aortic replacement, Hemiarch replacement, Total arch replacement, Aortic root replacement
Hsu 2020	Taiwan	Retrospective	3423	No follow up	Ascending aorta replacement, Total arch replacement, Aortic root replacement” Bentall”, Concomitant CABG, valve replacement-De Bakey type II dissection
Kawahito 2018	Japan	Retrospective	1026	5 ± 4.7 years	Ascending aorta/Valsalva replacement-aortic valve replacement-Partial or total arch replacement-Concomitant CABG
Ohnuma 2016	Japan	Retrospective	5175	No follow up	Aortic valve replacement, Total arch replacement, David procedure, Bentall procedure, and coronary bypass artery graft [CABG]
Omura 2017	Japan	Retrospective	345	4.3 ± 3.4 years	Ascending aorta replacement-Hemi arch replacement-Total arch replacement-Aortic root procedure (Bentall or reimplantation)-Coronary bypass grafting-
Suenaga 2016	Japan	Retrospective	80	6.8 ± 2.8 years and was 100% complete	Ascending aortic replacement (mainly), Total arch replacement, Aortic root replacement
Tochii 2016	Japan	Retrospective	158	3.4 ± 3.0 years and was 100% complete	Ascending aortic replacement, Total arch replacement, Root replacement
Suzuki 2019	Japan	Retrospective	319	3.8 (4.8) years in the older group and 4.9 (5.2) in the younger group.	Ascending aortic replacement, Hemiarch replacement, Total arch replacement, Root replacement
Rylski 2011	Germany	Prospective	464	no long-term follow-up.	Ascending aortic replacement, Hemiarch replacement, Total arch replacement, David operation, Yacoub operation
Benedetto 2021	England	Retrospective	3680	No follow up	Aortic Arch Replacement-Root Replacement-Concomitant CABG
Chavaron 2006	France	Retrospective	217	No follow up	Bentall procedure in 67 patients (31%) and an aortic valve replacement with an aortic tube in 4 cases (1.9%). aortic arch procedure was performed in 57 patients (26.4%): hemi arch in 51 patients (23.6%), and total arch replacement 6 patients (2.8%).CABG was performed in 14 patients (6.5%)
Goda 2010	Japan	Retrospective	301	No follow up	Ascending aortic replacement, Total arch replacement-Aortic root replacement (Bentall operation), Reimplantation of the aortic valve (David operation), Concomitant procedures (Coronary artery bypass grafting -Bypass to lower extremities, Probe Laparoscopy-Bypass to the superior mesenteric artery, Atrial septal defect closure)
Trimarchi 2010	USA, Germany, Italy, Spain	Retrospective	936	NR	Surgical management, Medical management, Percutaneous stenting or fenestration.
Chen 2022	Taiwan& Mongolia	Retrospective	123	58.25 (37.75) months	Ascending aortic grafting, Hemiarch replacement, Aortic valve repair
Igarashi 2020	Japan	Retrospective	199		Ascending aortic replacement, Hemiarch replacement, Total rch replacement, Root replacement
Shimamura 2018	Japan	Retrospective	300	31.7 ± 25.2 months	Hemiarch replacement, Total arch replacement

### Risk of bias assessment

Applying the NIH tool to our observational studies, 22 reports were of good quality, while five were of fair quality due to low follow-up duration (Supplementary Table 3).

### Octogenarians’ outcomes

**Fig. 1:**
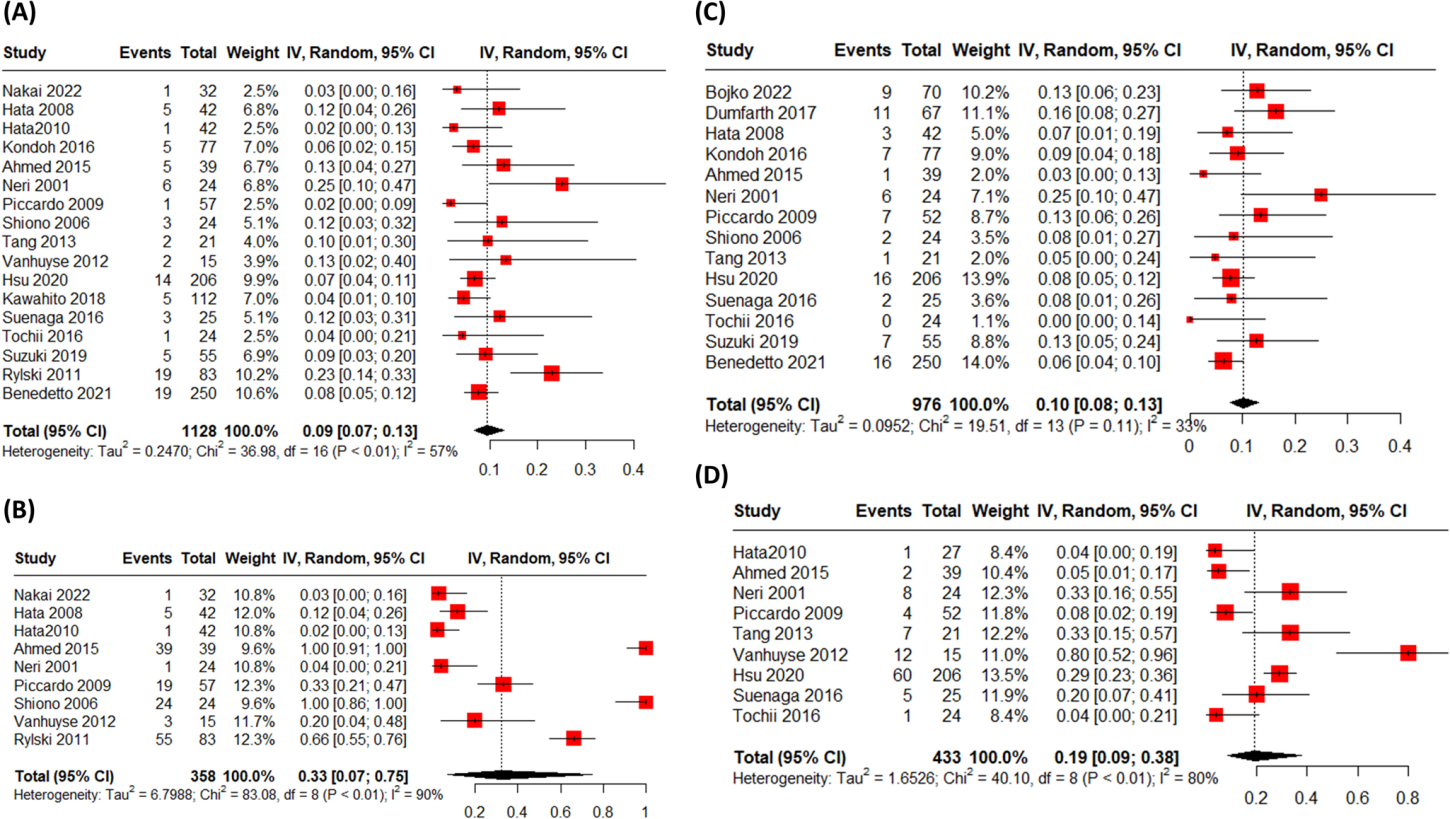
Outcomes of surgical repair in octogenarians with acute type A aortic dissection. **A** The rate of re-exploration in octogenarians treated with surgical repair for acute type A aortic dissection was 0.09 (95% CI: [0.07, 0.13]) based on the combination of data from 17 studies. **B** The rate of antegrade cerebral perfusion was 0.33 (95% CI: [0.07, 0.75]). **C** The rate ofstroke, derived from 14 studies, was 0.10 (95% CI: [0.08, 0.13]). **D** The rate of respiratory failure, combining data from 9 studies, was 0.19 (95% CI: [0.09, 0.38]).

Combining all 17 studies reporting re-exploration data of octogenarians treated with surgical repair for acute type A aortic dissection, The rate of re-exploration was rate 0.09 (95% CI: [0.07, 0.13]) in octogenarians treated with surgical repair for acute type A aortic dissection. (Fig. 1A). Regarding antegrade cerebral perfusion, the rate was 0.33 (95% CI: [0.07, 0.75]) (Fig. 1B), while the rate of stroke arising from the 14 studies was 0.10 (95% CI: [0.08, 0.13]) (Fig. 1C). About renal failure, the rate of events combining the 19 studies reporting this data was 0.10 (95% CI: [0.07, 0.15]) (supplementary Figure 2), while the rate of respiratory failure combining the 9 studies reporting this outcome was 0.19 (95% CI: [0.09, 0.38]) (Fig. 1D).

**Fig. 2:**
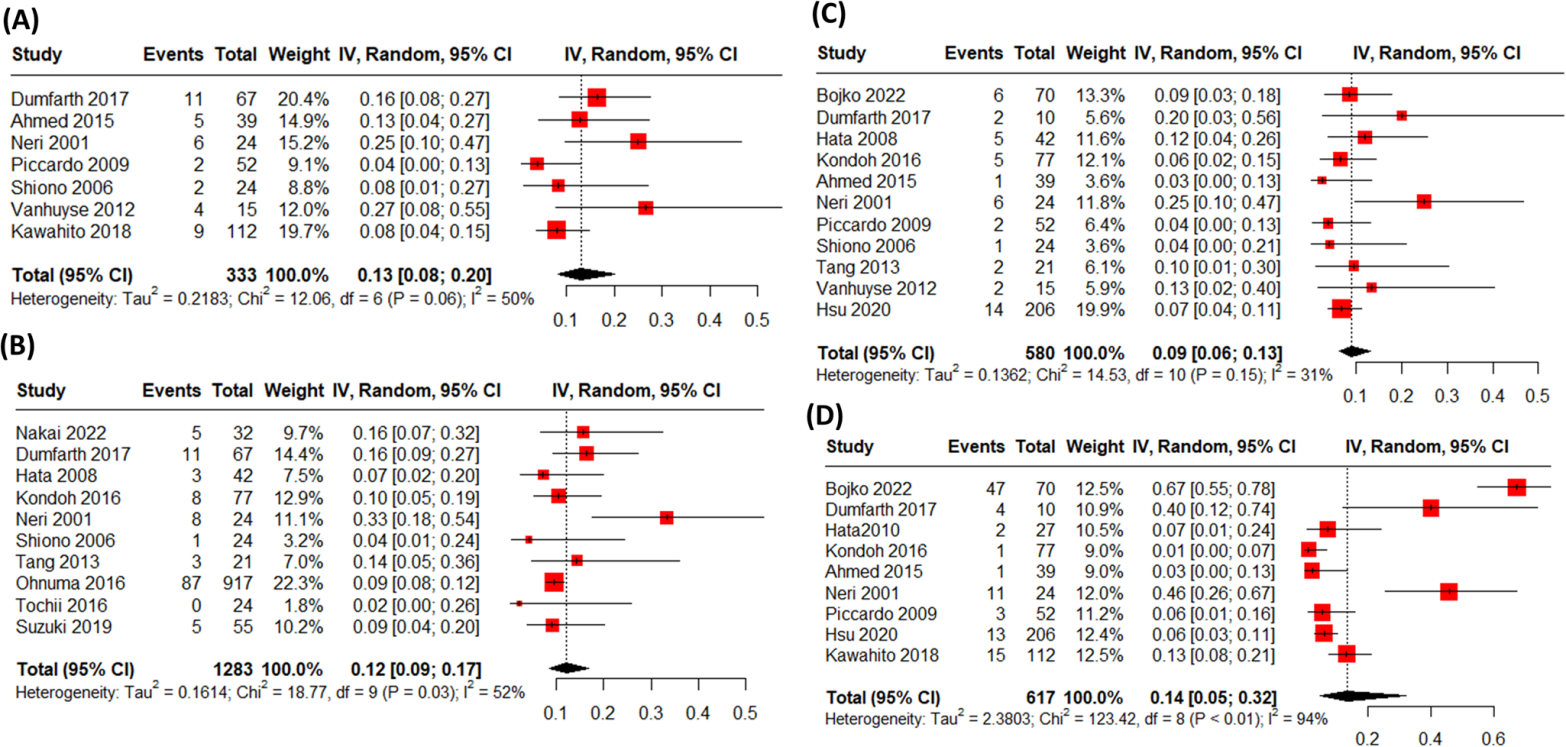
Complications following surgical repair in octogenarians with acute type A aortic dissection. **A** The rate of multi-organ failure was 0.13 (95% CI: [0.08, 0.20]), based on data from 10 studies. **B** The rate of tracheostomy was 0.12 (95% CI: [0.09, 0.17]), as reported in 10 studies. **C** The rate of bleeding events was 0.09 (95% CI: [0.06, 0.13]), combining data from 11 studies. **D** The rate of low cardiac output syndrome was 0.14 (95% CI: [0.05, 0.32]), derived from 9 studies.

For multi-organ failure, the rate was 0.13 (95% [CI: 0.08, 0.20]) (Fig. 2A). Combining all 10 studies reporting tracheostomy data for octogenarian patients, the rate was 0.12 (95% CI: [0.09, 0.17]) (Fig. 2B), while the rate of bleeding events arising from the 11 studies was 0.09 (95% CI: [0.06, 0.13]) (Fig. 2C). Regarding the low cardiac output syndrome, the pooled rate combining 9 studies was 0.14 (95% CI: [0.05, 0.32]) (Fig. 2D).

**Fig. 3:**
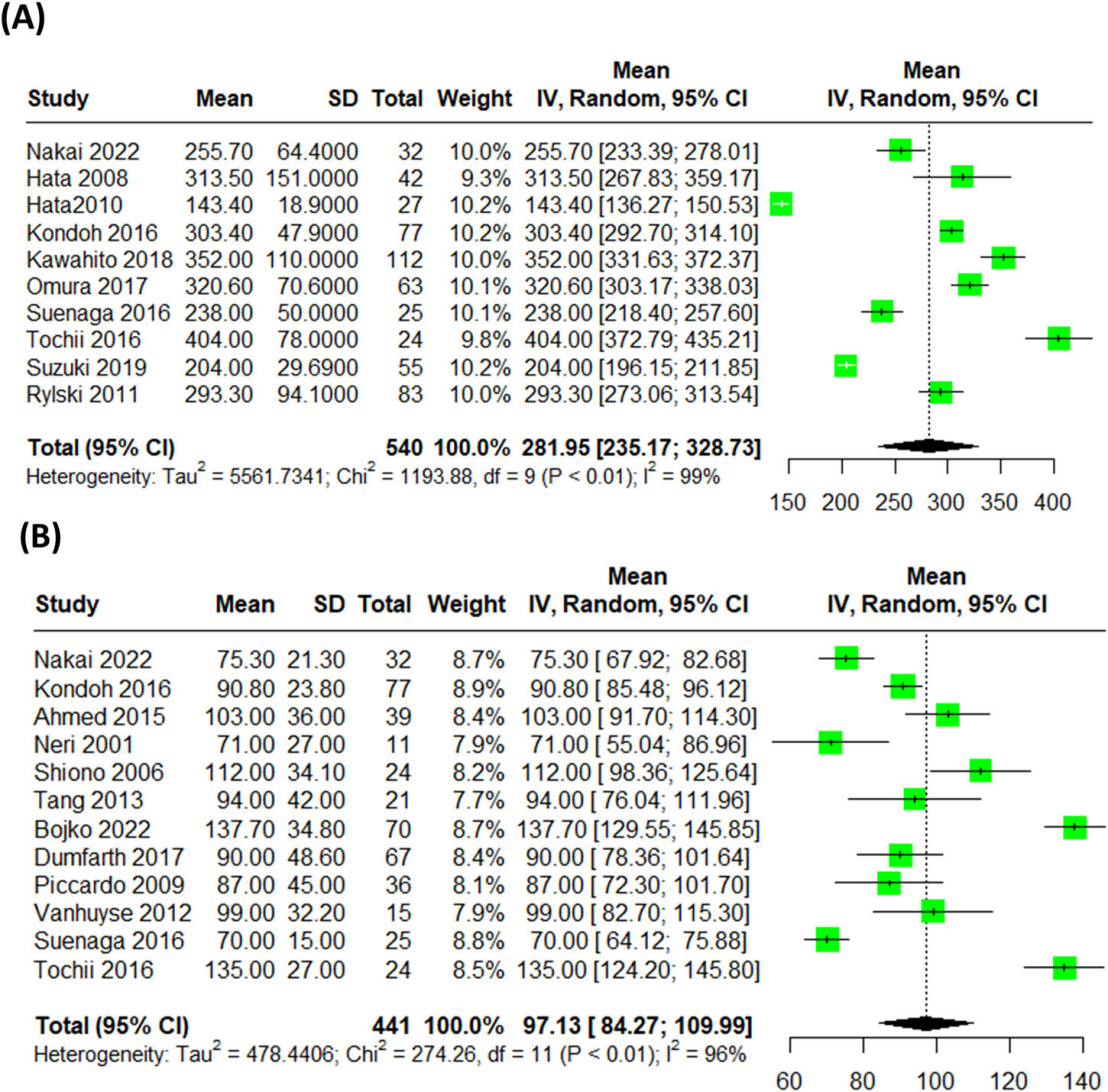
Surgical time metrics in octogenarians with acute type A aortic dissection. **A** The pooled mean operation time, reported in 10 studies, was 281.95 min (95% CI: [235.17, 328.73]). **B** The pooled mean myocardial ischemic time was 97.13 min (95% CI: [84.27, 109.99]).

Combining all 17 studies reporting Cardiopulmonary bypass time (min) of the octogenarians treated with surgical repair for acute type A aortic dissection, the pooled mean was (Mean = 161.89 (95% CI: 146.93, 176.85)) (Supplementary Fig. 3). About the operation time arising from the 10 studies reporting this outcome was (Mean = 281.95 (95% CI: 235.17, 328.73)) (Fig. 3A). Regarding the myocardial ischemic time, the pooled mean difference of was (Mean = 97.13 (95% CI: 84.27, 109.99)) (Fig. 3B).

### Octogenarians vs. septuagenarians

**Fig. 4:**
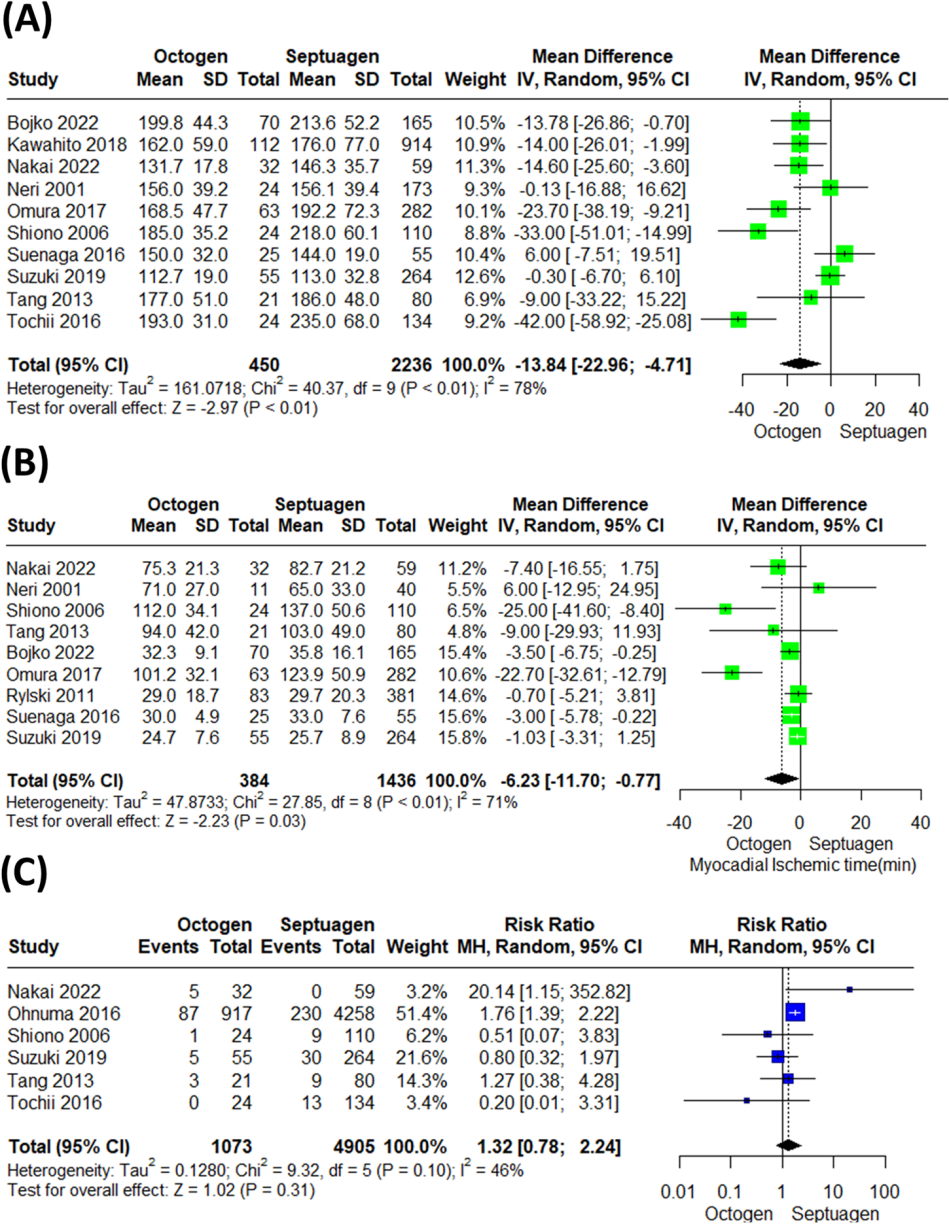
Comparative analysis of surgical outcomes between octogenarians and septuagenarians with acute type A aortic dissection. **A** Cardiopulmonary bypass surgery time was significantly shorter in octogenarians compared to septuagenarians (MD = −13.84, 95% CI: [−22.96, −4.71]). **B** Myocardial ischemic time was also shorter in the octogenarian group (MD = −6.23, 95% CI: [−11.70, −0.77]). **C** There was no significant difference in the rate of tracheostomy between the two groups (RR = 1.32, 95% CI: [0.78, 2.24]).

**Fig. 5:**
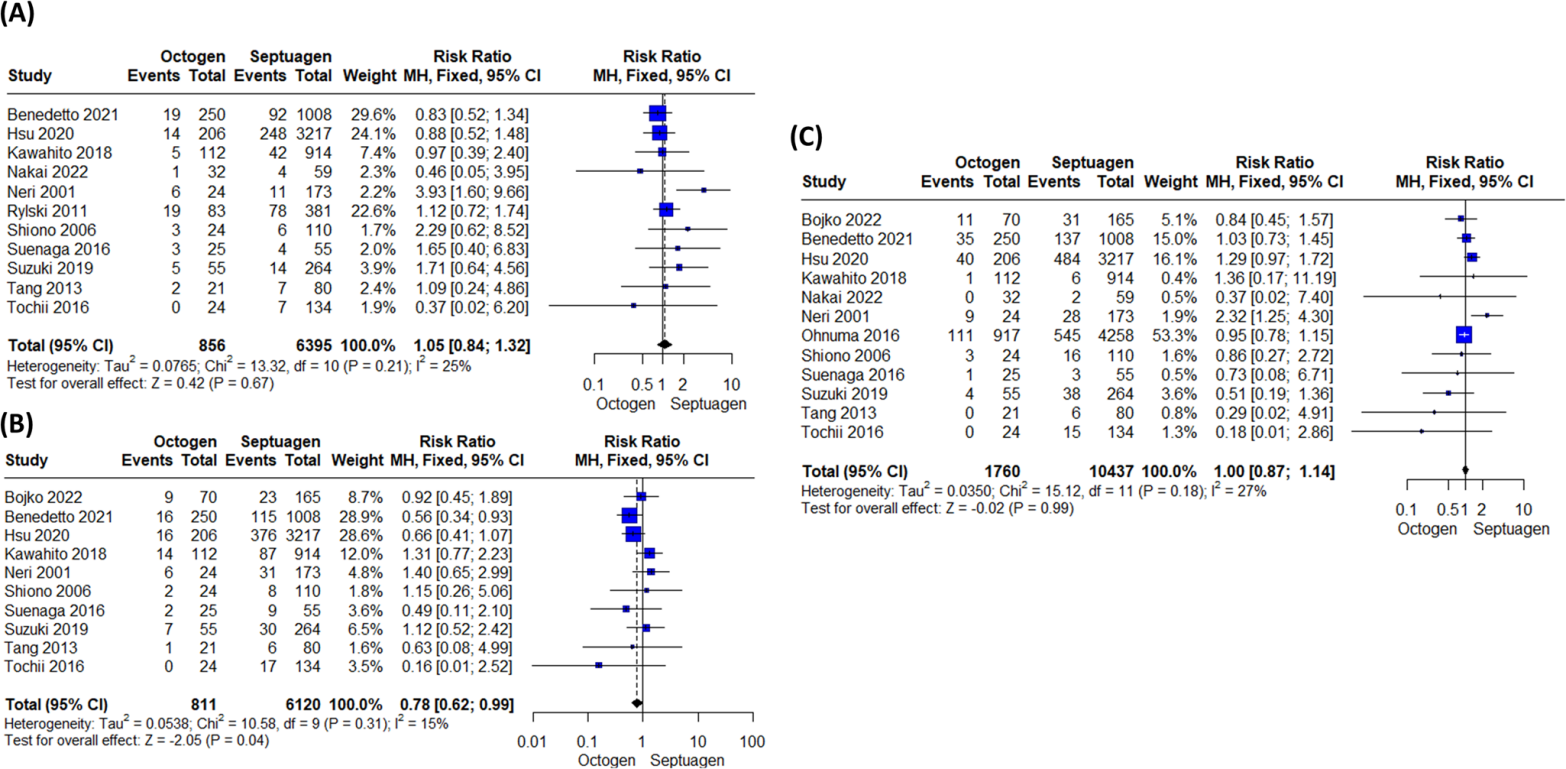
Comparative analysis of postoperative complications between octogenarians and septuagenarians with acute type A aortic dissection. **A** There was no significant difference in the rate of re-exploration between octogenarians and septuagenarians (RR = 1.05, 95% CI: [0.84, 1.32]). **B** The rate of stroke was also not significantly different between the two groups (RR = 0.78, 95% CI: [0.62, 0.99]). **C** Similarly, the rate of renal failure showed no significant difference between octogenarians and septuagenarians (RR = 1.00, 95% CI: [0.87, 1.14]).

The cardiopulmonary bypass surgery time and myocardial ischemic time were significantly shorter in the octogenarian group than in the younger group [(MD = −13.84 (95% CI: −22.96, −4.71) (Fig. 4A), (MD = −6.23 (95% CI: −11.70, −0.77)) (Fig. 4B); respectively]. In terms of tracheostomy (RR = 1.32 (95% CI: 0.78, 2.24)) (Fig. 4C), antegrade cerebral perfusion (RR = 0.94 (95% CI: 0.82, 1.08)) (Supplementary Fig. 4), re-exploration (RR = 1.05 (95% CI: 0.84, 1.32)) (Fig. 5A), Stroke (RR = 0.78 (95% CI: 0.62, 0.99)) (Fig. 5B), and renal failure (RR = 1.00 (95% CI: 0.87, 1.14)) (Fig. 5C), were not significantly different in both groups. However, the risk of respiratory complications in octogenarians were significantly higher than in septuagenarians (RR = 1.60 (95% CI: 1.31, 1.96)) (Supplementary Fig. 5).

### Heterogeneity, sensitivity analyses, and publication bias

No evidence of publication bias was observed for all our single and double-arm analysis outcomes by visual inspection of the funnel plots and confirmed by the Egger test except in single-arm analysis: cardiopulmonary bypass time (*p* = 0.001), operation time (*p* = 0.02). Further details can be found at (Supplementary Figs. 6 to 18). Moreover, most of our analyses had substantial heterogeneity, and whenever a substantial heterogeneity was observed a leave-one-out test was performed. High heterogeneity was observed in single-arm analysis in re-exploration, antegrade cerebral perfusion, respiratory failure, multi-organ failure, tracheostomy, low cardiac output syndrome, operation time (min), and myocardial ischemic time (min). In double arm analysis, cardiopulmonary bypass surgery time and myocardial ischemic time were solved by leave one out analysis except for antegrade cerebral perfusion. Further details about leave one out analysis can be found at (Supplementary Figs. 19 to 25).

## Discussion

The results of this meta-analysis demonstrated that octogenarians undergoing surgical repair for type A aortic dissection have a statistically significant difference in cardiopulmonary bypass surgery time, respiratory complications, and systemic cardiac arrest time when compared to septuagenarians; however, there was no statistically significant difference in cross-clamp time, myocardial ischemic time, tracheostomy, antegrade cerebral perfusion, neurological complications, stroke, renal failure, bleeding, and re-exploration.

For octogenarians patients with acute type A aortic dissection, a simple and quick procedure may be favorable^42,45^. The time required for cardiopulmonary bypass operation was shorter in octogenarians than in septuagenarians. In many previous studies, prolonged cardiac bypass time was related to increased hospital mortality^45,46^ Octogenarians had more restricted dissection and were less likely to undergo long procedures. Additionally, age reduces the likelihood of aortic dissection extension to the supra-aortic arteries and abdominal aorta^47^. In a cohort study, It is shown that reducing CPB times by executing a less invasive treatment such as ascending aorta replacement with an interposition graft was safe and effective^42^. Ghazy et al. ^48^ referred to this as a “defense strategy,” and they established that when older patients are handled in this manner, their health-related quality of life (HR-QOL) improves. It is suggested to use an approach that minimizes CPB operation time while reducing the life-threatening consequences of TAAD.

The repair procedure was carried out with an open technique during hypothermic circulatory arrest to induce cardiac arrest. In octogenarians, the time of systemic cardiac arrest was much shorter. According to a study, individuals who have a longer systemic cardiac arrest period have more difficult aortic surgical procedures and, as a result, a much longer surgical duration. By performing a less invasive procedure, circulatory arrest times in an elderly cohort can be reduced^43^. Hypothermic circulatory arrest, on the other hand, can result in abdominal organ dysfunction such as ischemia-reperfusion damage, and coagulation, nervous system, and kidney dysfunctions. Moreover, the occurrence of these dysfunctions is proportional to the length of hypothermic circulatory arrest^49–52^. So, if surgeons cannot complete high-quality complex surgery as soon as possible during safe operational time window, patients may be left with serious complications. So, they need relatively advanced skills, much experience, and excellent psychological quality.

Respiratory complications were more in octogenarians than in septuagenarians. This might be due to a decrease in immunity as age progresses^53^. Octogenarians lack the same physiological reserve as younger patients and do not recover as quickly from surgical complications^54^. All these factors are beyond the control of the surgeon. However, postoperative care that did not include adequate lung protective ventilation, the prone position, or restrictive fluid-volume management may have exacerbated postoperative respiratory dysfunction^55^. There are currently no studies on the prevention of respiratory complications after surgery for type A aortic dissection using preventive and therapeutic antibiotic therapy^56^.

Octogenarians had a lower 5-year survival rate than septuagenarians, most likely due to the age gap between these two groups. Piccardo et al. ^27,57^ observed poor survival with a hospital mortality rate of more than 40% and a 5-year overall survival of less than 50% after AAAD surgery in a series of single institutional and multicenter investigations in octogenarians. One of the key factors of hospital mortality may be surgeon experience and surgical procedures performed. In a Japanese study, the in-hospital mortality and 5-year overall survival rates in octogenarians with type A aortic dissection (*n* = 112) were 6.3 and 57.1%, respectively^21^. A future study is required to identify whether the cause of death reflects patient history characteristics, in-hospital parameters, or in-hospital complications.

Our results shows that no statistically difference between the two groups regarding neurological complications. However, a multicenter study by Piccardo et al. found that 43.8% of octogenarians presented with complicated dissection (neurologic deficit or shock)^27^. Preoperative neurological symptoms, consciousness abnormalities, CPR, hemodynamic instability, hypotension, and intubation of the patient upon hospital admission are all characteristics that are statistically associated with postoperative neurological complications attendance^58^. In Dumfarth’s study^59^, there was a significant correlation between preoperative and postoperative neurological complications (25% vs. 11.8%, *p* = 0.022). So, advanced neuroprotective strategies during surgical treatment of AADA are required.

Our study has a number of limitations. First, all of the included studies are cohort studies that have their internal limitations. Second, the difference of sample size between the two groups makes it difficult to interpret the results. Third, the survival difference in this study should be evaluated with caution since it is restricted by the failure to differentiate aortic-related death rates from other causes of death. Fourth, our included studies did not include individuals aged 70 or older who did not have surgery due to the severity of their condition, a family choice, died on the way to the hospital, or other unexplained causes. Fifth, the wide range variability between cases in the extension of the pathology and type of surgical repair offered by the surgeon. Sixth, the substantial heterogeneity in our analysis. So, patient selection limits the generalizability of our study.

To better understand the clinical presentation, the effects of medical care, and the impact of this illness on long-term survival and quality of life in the elderly, future studies should analyse the experience of all octogenarians presenting with ATAAD, not only those who underwent surgery. Although postoperative patient status should be recognized as a key component of total surgical success, functional status and quality of life after surgery were not studied in this study. There is insufficient evidence to conduct a randomized comparison of isolated ascending aortic replacement vs complete arch replacement. While Hypothermia Circulatory Arrest has become a key aspect of arch surgery, the appropriate regulation of its duration is still being debated.

In conclusion, considering the fragility context among octogenarians, surgical repair of type A aortic dissection in octogenarians yields relatively lower complication rates, including re-exploration, stroke, and renal failure. Compared to septuagenarians, octogenarians experience shorter cardiopulmonary bypass and myocardial ischemic times. However, they face a significantly higher risk of respiratory complications, highlighting unique challenges in managing this elderly population surgically.

### Recommendations and practical implications

- It is crucial to prioritize individualized assessment and comprehensive care for octogenarians undergoing surgical repair.
- Close monitoring of indicators like multi-organ failure, tracheostomy necessity, bleeding events, and low cardiac output syndrome during postoperative care aims to sustain favorable outcomes in octogenarians.
- Prioritize respiratory care for octogenarians to reduce complications and improve surgical outcomes.

## Supplementary information

Supplementary information
