# Supplementary material for: Acute Type A aortic dissection surgical repair in Octogenarians: A meta-analysis
Source: NPJ Cardiovasc Health. 2024 Aug 31;1:16. doi: 10.1038/s44325-024-00007-9 (PMC12912317; doi:10.1038/s44325-024-00007-9)
Supplement: Supplementary file 1 — Supplementary information [file 44325_2024_7_MOESM1_ESM.pdf]

Appendix

Supplementary Figure 1: PRISMA Flow chart

Supplementary Figure 2: Forest plot of single octogenarian arm analysis of renal failure.

Supplementary Figure 3: Forest plot of single octogenarian arm analysis of Cardiopulmonary bypass time (min)

Supplementary Figure 4: Forest plot of double arm analysis of antegrade cerebral perfusion

Supplementary Figure 5: Forest plot of double arm analysis of respiratory complications

Supplementary Figure 6: Funnel plot of single arm analysis of bleeding

Supplementary Figure 7: Funnel plot of single arm analysis of myocardial ischemic time.

Supplementary Figure 8: Funnel plot of single arm analysis of re-exploration

Supplementary Figure 9: Funnel plot of single arm analysis of renal failure

Supplementary Figure 10: Funnel plot of single arm analysis of stroke

Supplementary Figure 11: Funnel plot of single arm analysis of tracheostomy

Supplementary Figure 12: Funnel plot of single arm analysis of cardiopulmonary bypass time

Supplementary Figure 13: Funnel plot of single arm analysis of circulatory arrest time

Supplementary Figure 14: Funnel plot of single arm analysis of operation time

Supplementary Figure 15: Funnel plot of double arm analysis of cardiopulmonary bypass time

Supplementary Figure 16: Funnel plot of double arm analysis of re-exploration

Supplementary Figure 17: Funnel plot of double arm analysis of renal failure

Supplementary Figure 18: Funnel plot of double arm analysis of stroke

Supplementary Figure 19: Leave on out test of single arm analysis of tracheostomy
Supplementary Figure 20: Leave on out test of single arm analysis of low cardiac output syndrome.

Supplementary Figure 21: Leave on out test of single arm analysis of operation time (min).

Supplementary Figure 22: Leave on out test of single arm analysis of myocardial ischemic time (min).

Supplementary Figure 23: Leave on out test of double arm analysis of cardiopulmonary bypass surgery time (min).

Supplementary Figure 24: Leave on out test of double arm analysis of myocardial ischemic time (min).

Supplementary Figure 25: Leave on out test of double arm analysis of antegrade cerebral perfusion.

Supplementary Figure 26: Leave on out test of double arm analysis of respiratory failure.

Supplementary Table 1: PRISMA checklist

Supplementary table 2: Baseline characteristics of included studies.

Supplementary Table 3: NIH tool for assessing risk of bias.

Supplementary Figure 1: PRISMA Flow chart

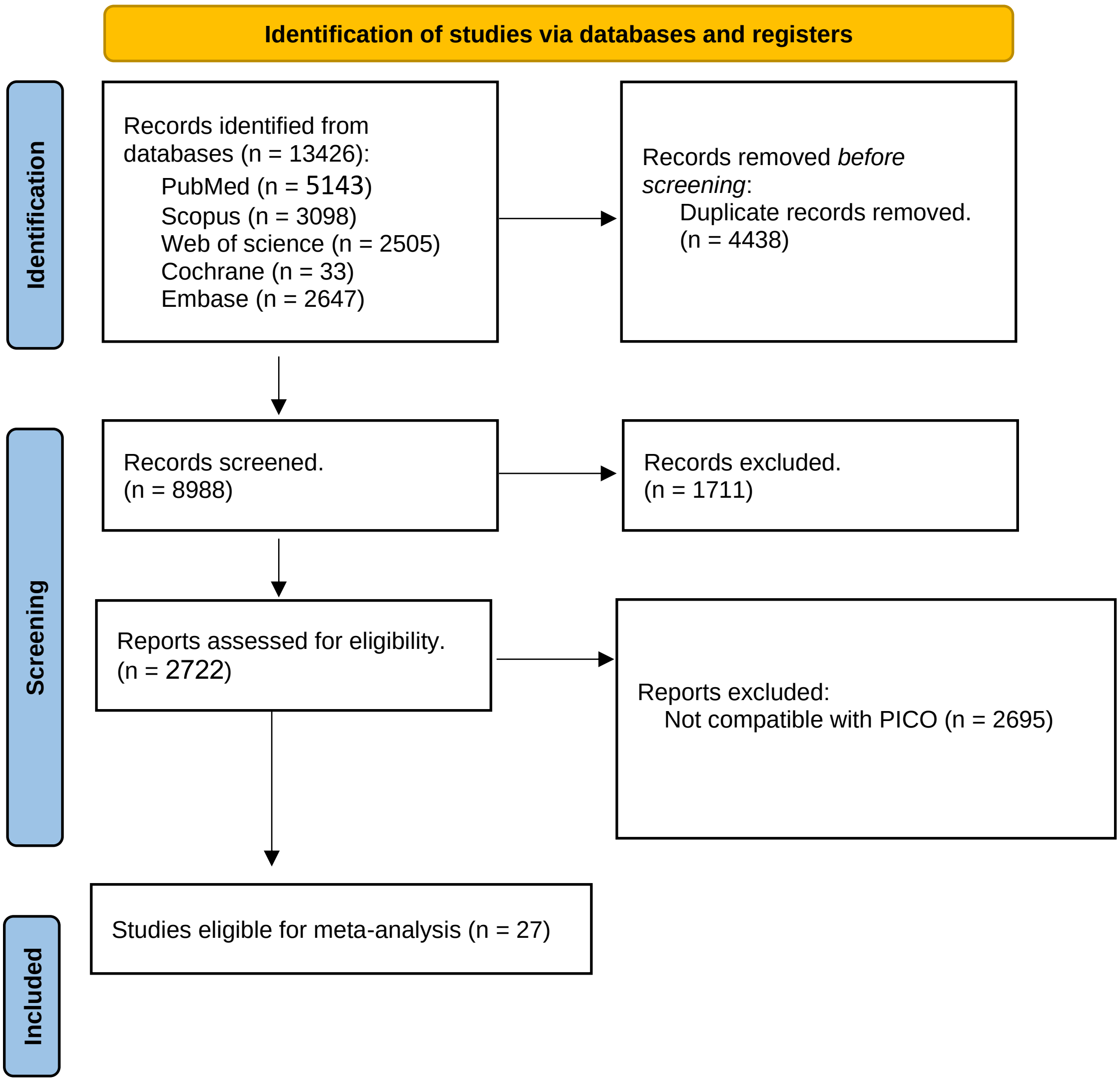

Supplementary Figure 2: Forest plot of single octogenarian arm analysis of renal failure.

Study	Events	Total	Weight	IV, Random, 95% CI
Bojko 2022	11	70	7.1%	0.16 [0.08; 0.26]
Nakai 2022	1	59	3.1%	0.02 [0.00; 0.09]
Dumfarth 2017	4	67	5.8%	0.06 [0.02; 0.15]
Hata 2008	6	42	6.3%	0.14 [0.05; 0.29]
Hata2010	1	27	3.0%	0.04 [0.00; 0.19]
Kondoh 2016	2	77	4.5%	0.03 [0.00; 0.09]
Ahmed 2015	5	39	6.1%	0.13 [0.04; 0.27]
Neri 2001	9	24	6.5%	0.38 [0.19; 0.59]
Piccardo 2009	14	52	7.3%	0.27 [0.16; 0.41]
Shiono 2006	3	24	5.1%	0.12 [0.03; 0.32]
Tang 2013	1	21	3.0%	0.05 [0.00; 0.24]
Hsu 2020	40	206	8.1%	0.19 [0.14; 0.25]
Kawahito 2018	1	112	3.1%	0.01 [0.00; 0.05]
Ohnuma 2016	111	917	8.3%	0.12 [0.10; 0.14]
Omura 2017	1	23	3.0%	0.04 [0.00; 0.22]
Suenaga 2016	1	25	3.0%	0.04 [0.00; 0.20]
Tochii 2016	1	24	3.0%	0.04 [0.00; 0.21]
Suzuki 2019	4	55	5.8%	0.07 [0.02; 0.18]
Benedetto 2021	35	250	8.0%	0.14 [0.10; 0.19]
**Total (95% CI)**		**2114**	**100.0%**	**0.10 [0.07; 0.15]**

Heterogeneity: $Tau^2 = 0.5706$; $Chi^2 = 57.29$, df = 18 ($P < 0.01$); $I^2 = 69\%$

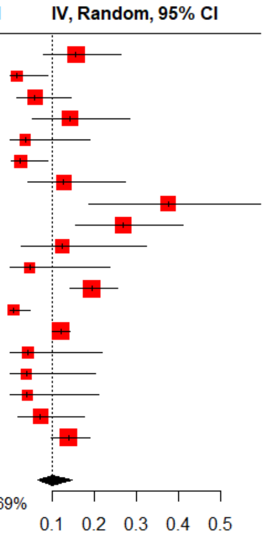

Supplementary Figure 3: Forest plot of single octogenarian arm analysis of Cardiopulmonary bypass time (min)

Study	Mean	SD	Total	Weight	Mean IV, Random, 95% CI
Bojko 2022	199.80	44.3000	70	6.1%	199.80 [189.42; 210.18]
Nakai 2022	131.70	17.8000	32	6.2%	131.70 [125.53; 137.87]
Dumfarth 2017	177.60	69.1000	67	5.8%	177.60 [161.05; 194.15]
Hata 2008	153.90	67.7000	42	5.6%	153.90 [133.43; 174.37]
Hata2010	82.80	11.8000	27	6.2%	82.80 [78.35; 87.25]
Kondoh 2016	176.30	49.7000	77	6.0%	176.30 [165.20; 187.40]
Ahmed 2015	163.00	47.0000	39	5.9%	163.00 [148.25; 177.75]
Neri 2001	156.00	39.2240	24	5.9%	156.00 [140.31; 171.69]
Piccardo 2009	182.00	81.0000	57	5.6%	182.00 [160.97; 203.03]
Shiono 2006	185.00	35.2000	24	5.9%	185.00 [170.92; 199.08]
Tang 2013	177.00	51.0000	21	5.5%	177.00 [155.19; 198.81]
Vanhuyse 2012	194.00	59.3000	15	5.0%	194.00 [163.99; 224.01]
Kawahito 2018	162.00	59.0000	112	6.1%	162.00 [151.07; 172.93]
Omura 2017	168.50	47.7000	63	6.0%	168.50 [156.72; 180.28]
Suenaga 2016	150.00	32.0000	25	6.0%	150.00 [137.46; 162.54]
Tochii 2016	193.00	31.0000	24	6.0%	193.00 [180.60; 205.40]
Suzuki 2019	112.70	19.0300	55	6.2%	112.70 [107.67; 117.73]
**Total (95% CI)**			**774**	**100.0%**	**161.89 [146.93; 176.85]**

Heterogeneity: $Tau^2 = 931.1339$; $Chi^2 = 1085.03$, df = 16 ($P < 0.01$); $I^2 = 99\%$

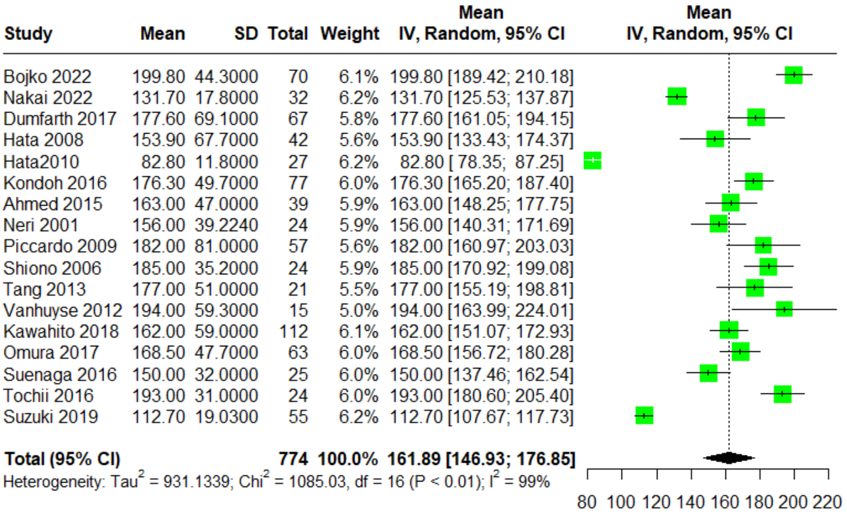

Supplementary Figure 4: Forest plot of double arm analysis of antegrade cerebral perfusion

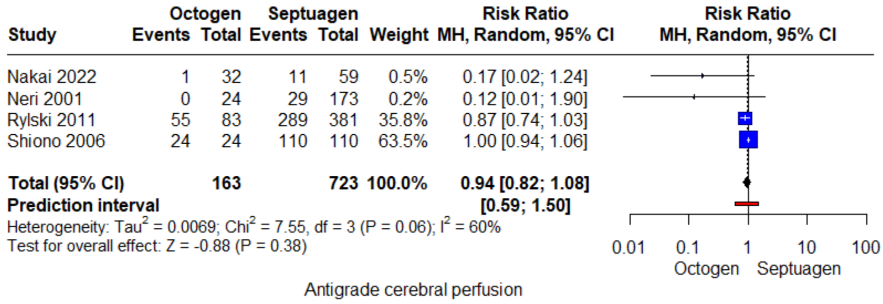

Supplementary Figure 5: Forest plot of double arm analysis of respiratory complications

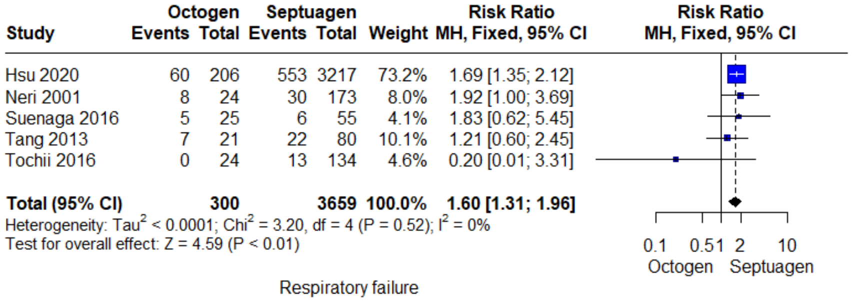

Supplementary Figure 6: Funnel plot of single arm analysis of bleeding

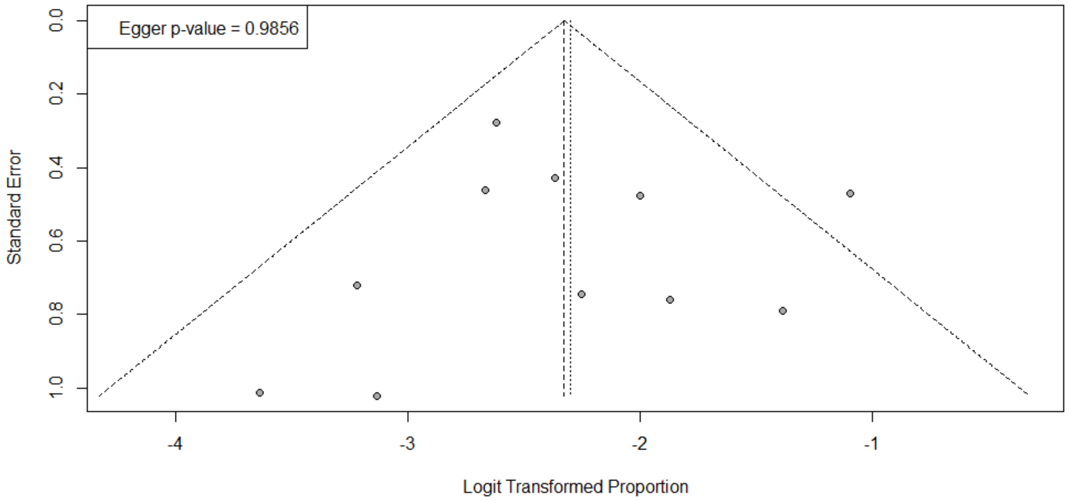

Supplementary Figure 7: Funnel plot of single arm analysis of myocardial ischemic time.

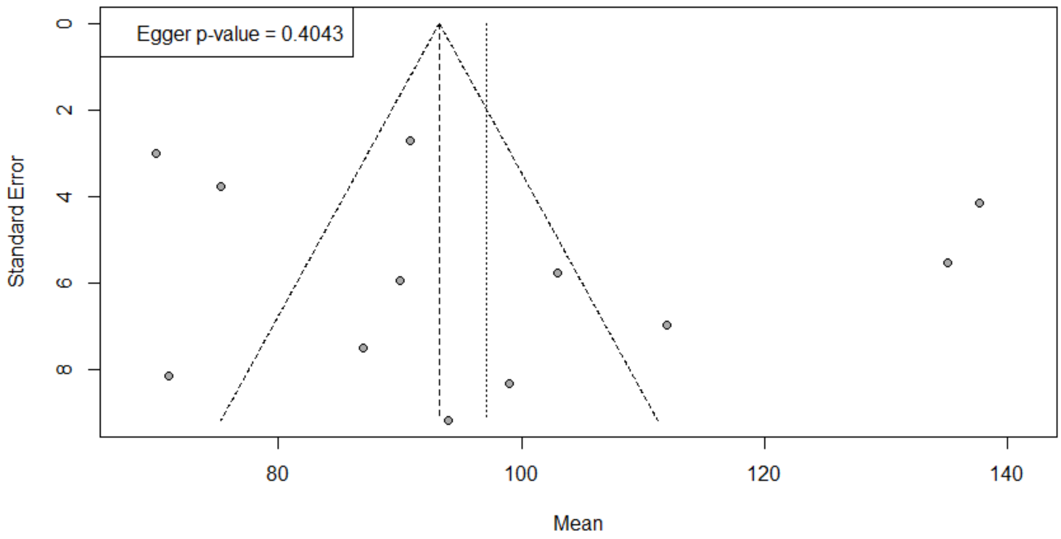

Supplementary Figure 8: Funnel plot of single arm analysis of re-exploration

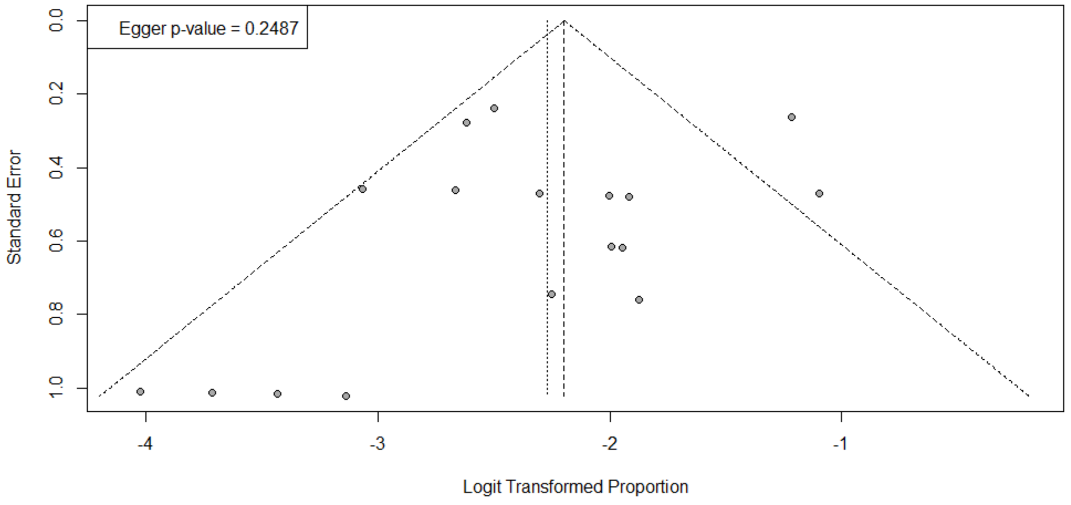

Supplementary Figure 9: Funnel plot of single arm analysis of renal failure

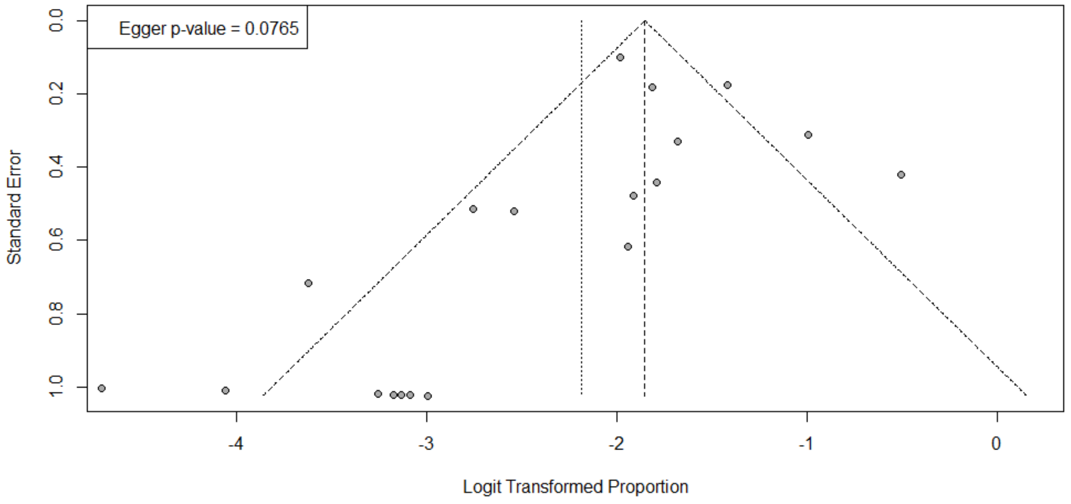

Supplementary Figure 10: Funnel plot of single arm analysis of stroke

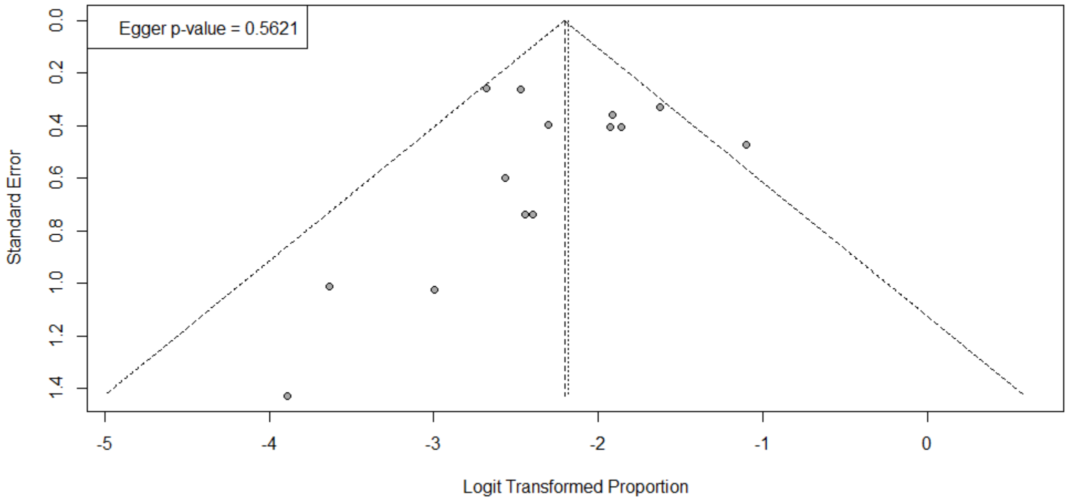

Supplementary Figure 11: Funnel plot of single arm analysis of tracheostomy

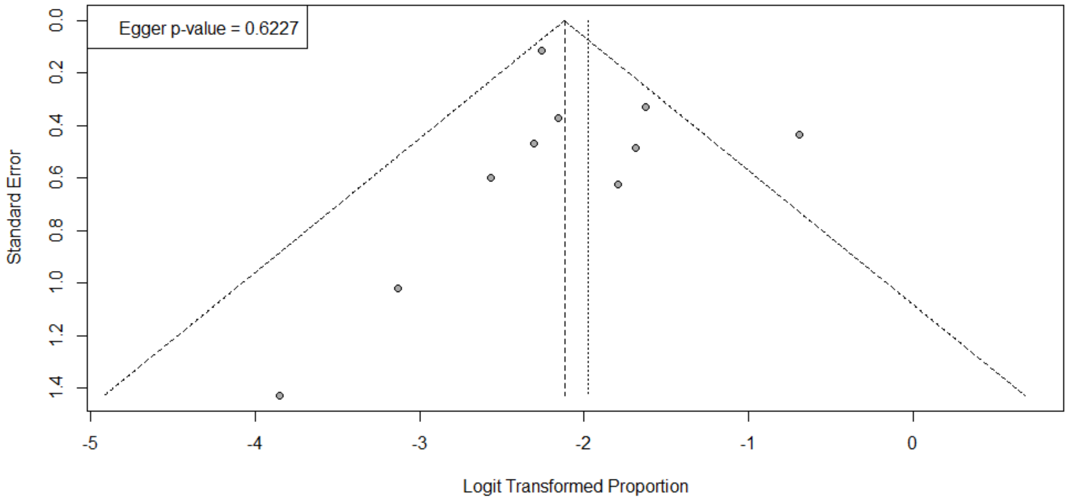

Supplementary Figure 12: Funnel plot of single arm analysis of cardiopulmonary bypass time

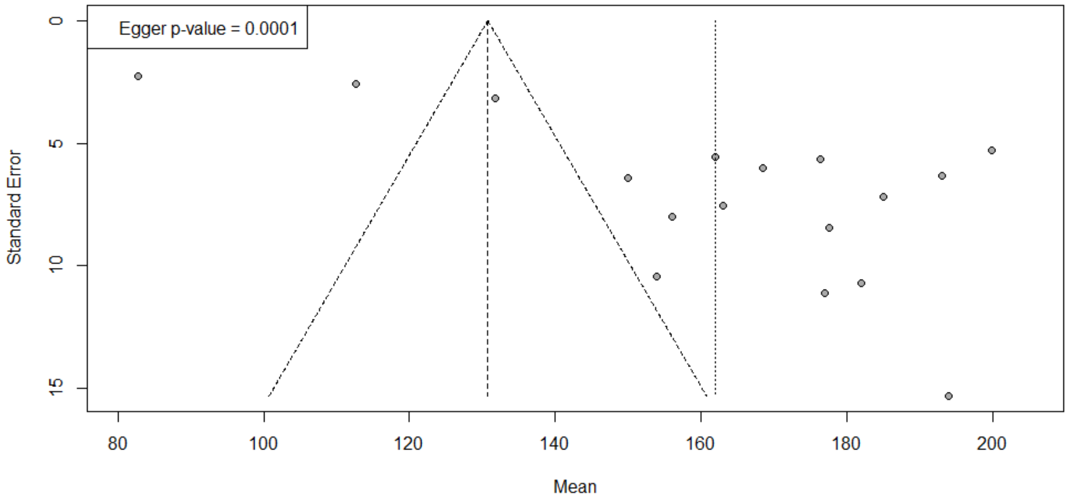

Supplementary Figure 13: Funnel plot of single arm analysis of circulatory arrest time

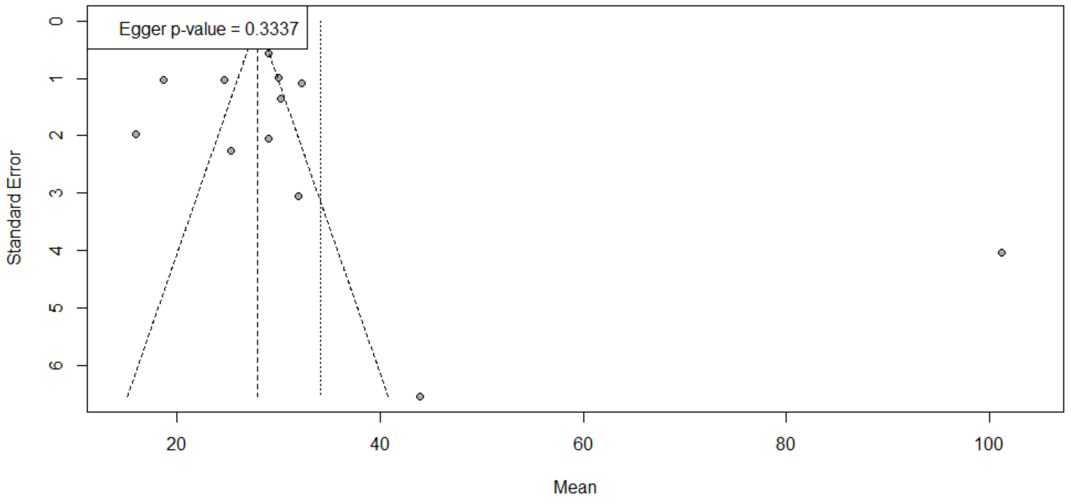

Supplementary Figure 14: Funnel plot of single arm analysis of operation time

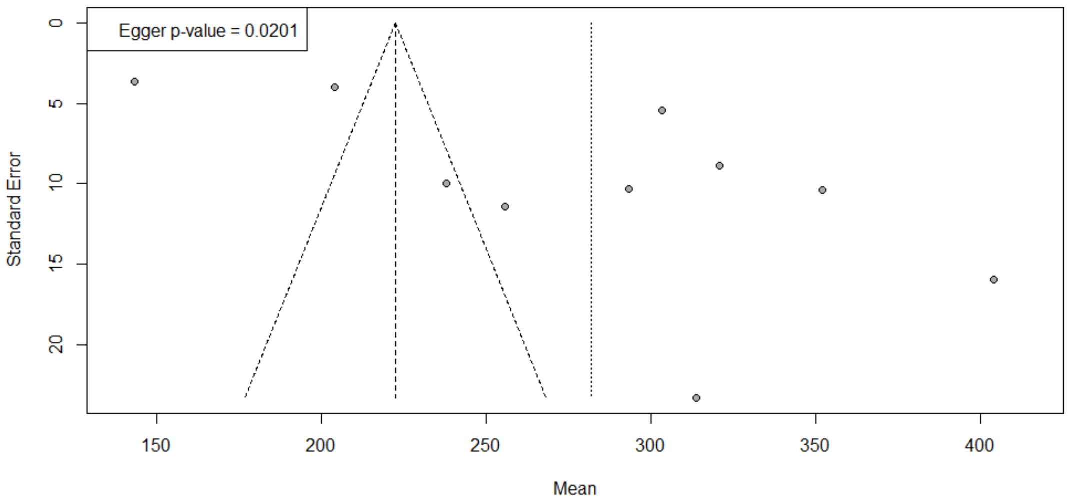

Supplementary Figure 15: Funnel plot of double arm analysis of cardiopulmonary bypass time

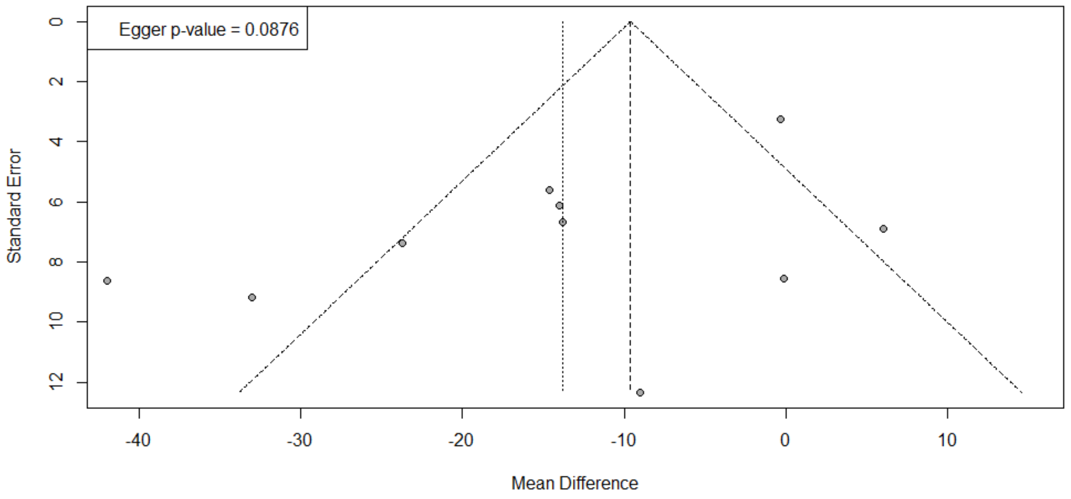

Supplementary Figure 16: Funnel plot of double arm analysis of re-exploration

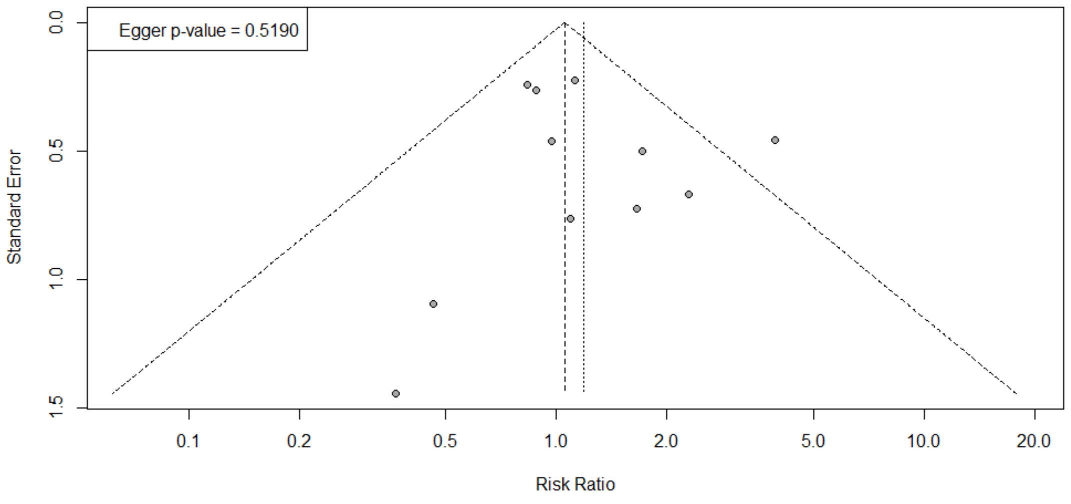

Supplementary Figure 17: Funnel plot of double arm analysis of renal failure

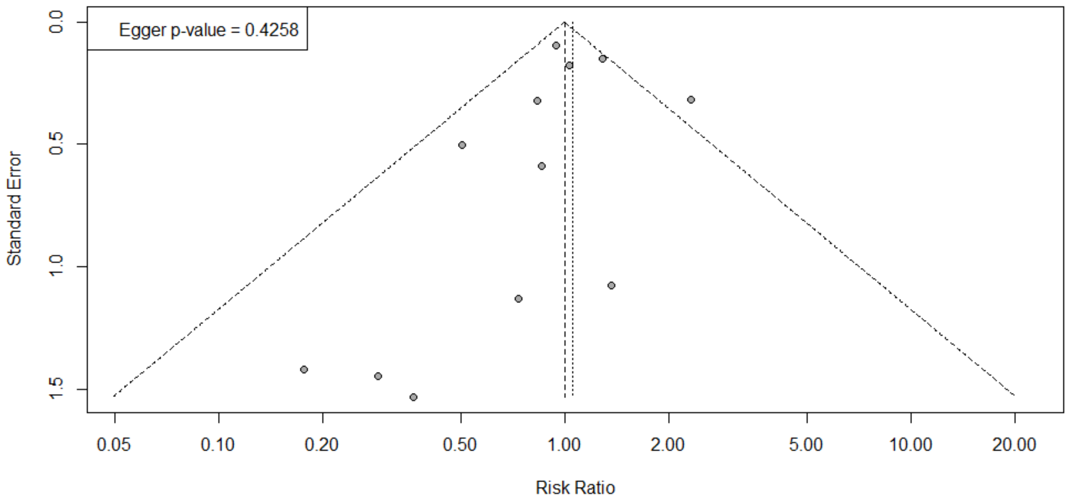

Supplementary Figure 18: Funnel plot of double arm analysis of stroke

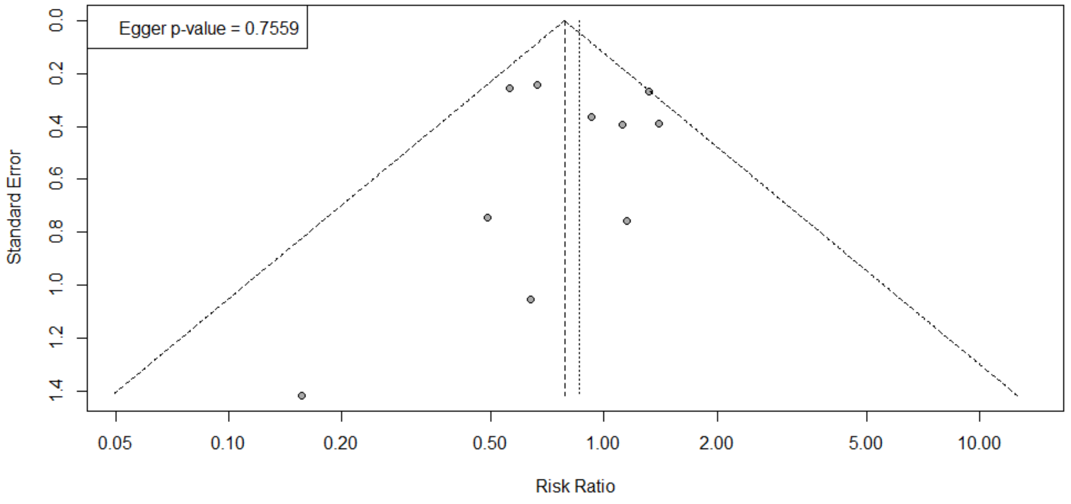

Supplementary Figure 19: Leave on out test of single arm analysis of tracheostomy.

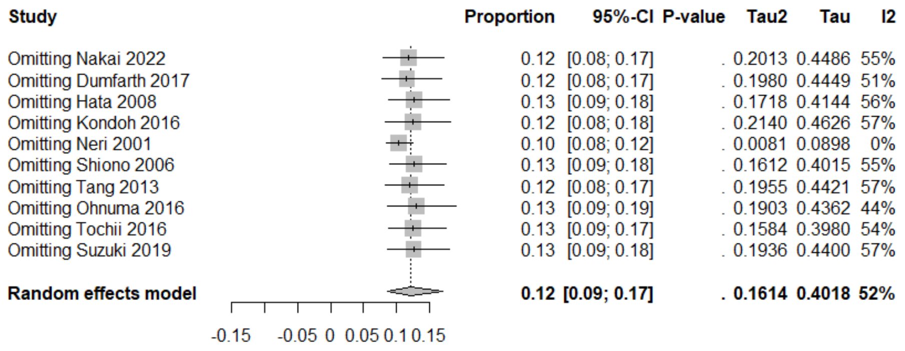

Supplementary Figure 20: Leave on out test of single arm analysis of low cardiac output syndrome.

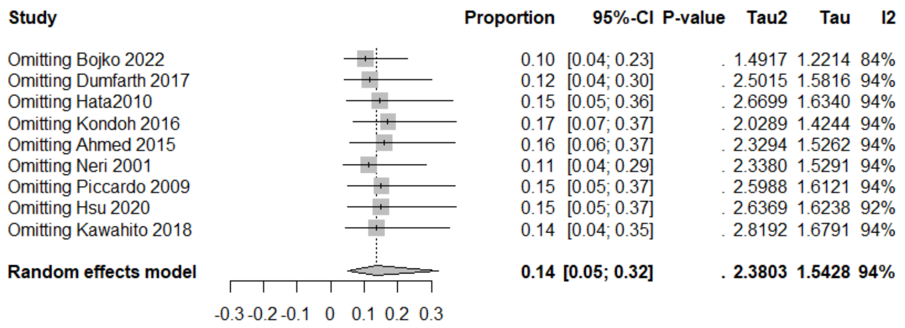

Supplementary Figure 21: Leave on out test of single arm analysis of operation time (min).

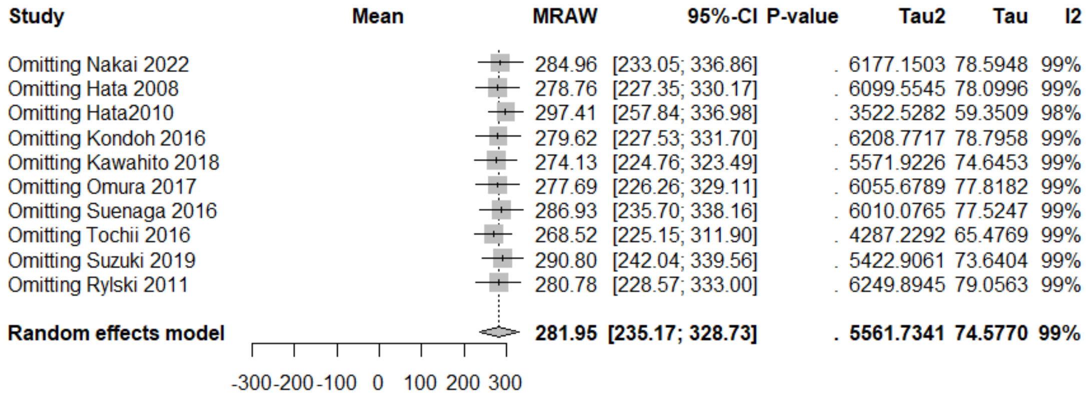

Supplementary Figure 22: Leave on out test of single arm analysis of myocardial ischemic time (min).

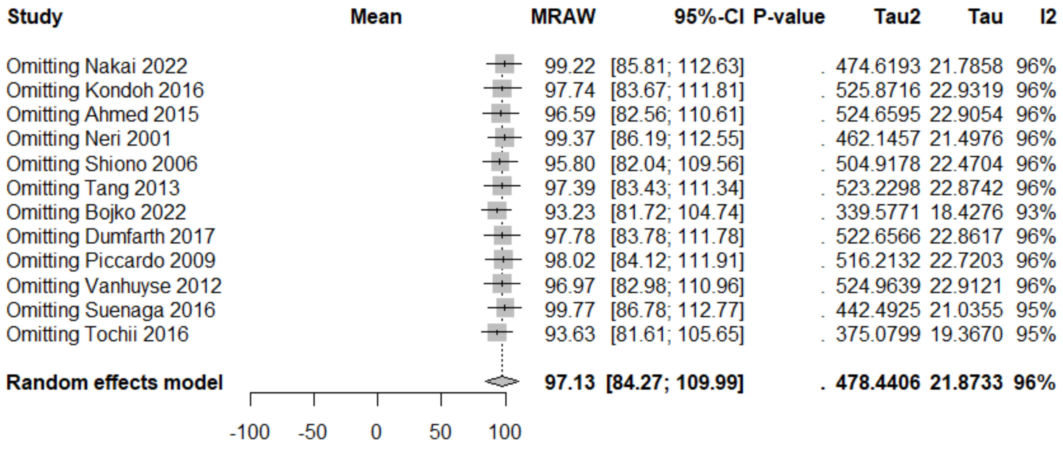

Supplementary Figure 23: Leave on out test of double arm analysis of cardiopulmonary bypass surgery time (min).

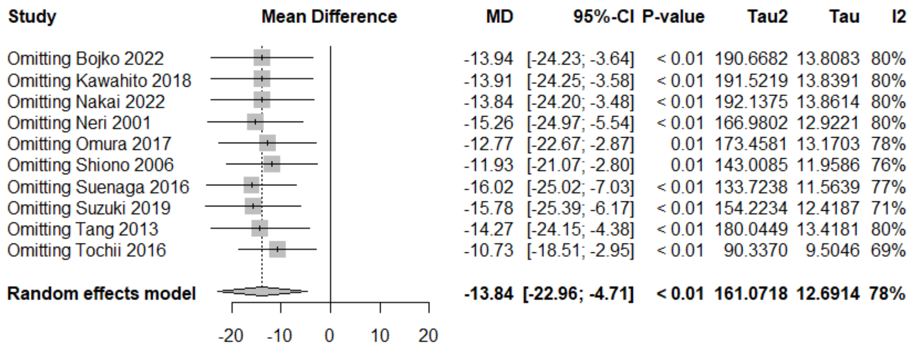

Supplementary Figure 24: Leave on out test of double arm analysis of myocardial ischemic time (min).

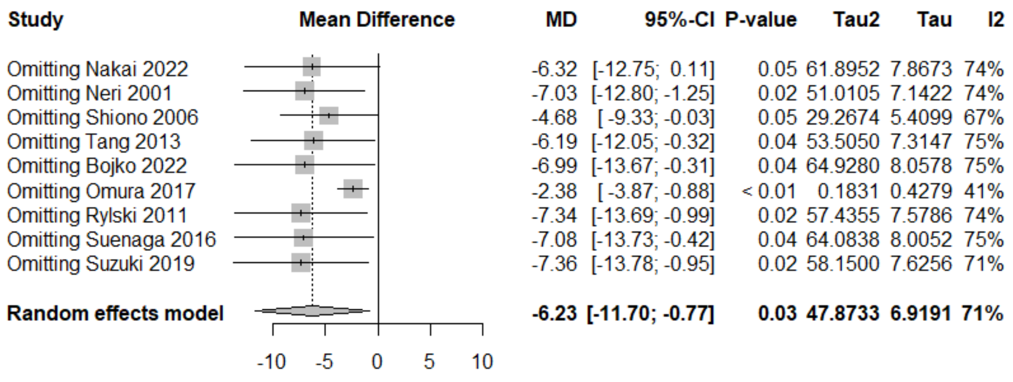

Supplementary Figure 25: Leave on out test of double arm analysis of antegrade cerebral perfusion.

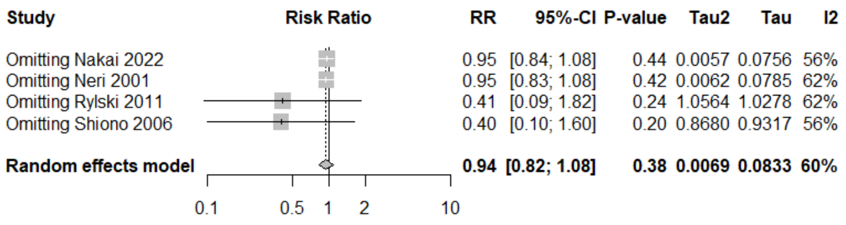

Supplementary Table 1: PRISMA checklist

Section and Topic	Item #	Checklist item	Location where item is reported
**TITLE**			
Title	1	Identify the report as a systematic review.	1
**ABSTRACT**			
Abstract	2	See the PRISMA 2020 for Abstracts checklist.	3
**INTRODUCTION**			
Rationale	3	Describe the rationale for the review in the context of existing knowledge.	5
Objectives	4	Provide an explicit statement of the objective(s) or question(s) the review addresses.	5
**METHODS**			
Eligibility criteria	5	Specify the inclusion and exclusion criteria for the review and how studies were grouped for the syntheses.	6
Information sources	6	Specify all databases, registers, websites, organisations, reference lists and other sources searched or consulted to identify studies. Specify the date when each source was last searched or consulted.	6
Search strategy	7	Present the full search strategies for all databases, registers and websites, including any filters and limits used.	6
Selection process	8	Specify the methods used to decide whether a study met the inclusion criteria of the review, including how many reviewers screened each record and each report retrieved, whether they worked independently, and if applicable, details of automation tools used in the process.	6
Data collection process	9	Specify the methods used to collect data from reports, including how many reviewers collected data from each report, whether they worked independently, any processes for obtaining or confirming data from study investigators, and if applicable, details of automation tools used in the process.	6
Data items	10a	List and define all outcomes for which data were sought. Specify whether all results that were compatible with each outcome domain in each study were sought (e.g. for all measures, time points, analyses), and if not, the methods used to decide which results to collect.	7
	10b	List and define all other variables for which data were sought (e.g. participant and intervention characteristics, funding sources). Describe any assumptions made about any missing or unclear information.	7
Study risk of bias assessment	11	Specify the methods used to assess risk of bias in the included studies, including details of the tool(s) used, how many reviewers assessed each study and whether they worked independently, and if applicable, details of automation tools used in the process.	7
Effect measures	12	Specify for each outcome the effect measure(s) (e.g. risk ratio, mean difference) used in the synthesis or presentation of results.	7
Synthesis methods	13a	Describe the processes used to decide which studies were eligible for each synthesis (e.g. tabulating the study intervention characteristics and comparing against the planned groups for each synthesis (item #5)).	7
	13b	Describe any methods required to prepare the data for presentation or synthesis, such as handling of missing summary statistics, or data conversions.	7
	13c	Describe any methods used to tabulate or visually display results of individual studies and syntheses.	7
	13d	Describe any methods used to synthesize results and provide a rationale for the choice(s). If meta-analysis was	7

Section and Topic	Item #	Checklist item	Location where item is reported
		performed, describe the model(s), method(s) to identify the presence and extent of statistical heterogeneity, and software package(s) used.	
	13e	Describe any methods used to explore possible causes of heterogeneity among study results (e.g. subgroup analysis, meta-regression).	7
	13f	Describe any sensitivity analyses conducted to assess robustness of the synthesized results.	7
Reporting bias assessment	14	Describe any methods used to assess risk of bias due to missing results in a synthesis (arising from reporting biases).	7
Certainty assessment	15	Describe any methods used to assess certainty (or confidence) in the body of evidence for an outcome.	7
**RESULTS**			
Study selection	16a	Describe the results of the search and selection process, from the number of records identified in the search to the number of studies included in the review, ideally using a flow diagram.	8
	16b	Cite studies that might appear to meet the inclusion criteria, but which were excluded, and explain why they were excluded.	8
Study characteristics	17	Cite each included study and present its characteristics.	8
Risk of bias in studies	18	Present assessments of risk of bias for each included study.	8
Results of individual studies	19	For all outcomes, present, for each study: (a) summary statistics for each group (where appropriate) and (b) an effect estimate and its precision (e.g. confidence/credible interval), ideally using structured tables or plots.	8
Results of syntheses	20a	For each synthesis, briefly summarise the characteristics and risk of bias among contributing studies.	9, 10, 11
	20b	Present results of all statistical syntheses conducted. If meta-analysis was done, present for each the summary estimate and its precision (e.g. confidence/credible interval) and measures of statistical heterogeneity. If comparing groups, describe the direction of the effect.	9, 10, 11
	20c	Present results of all investigations of possible causes of heterogeneity among study results.	9, 10, 11
	20d	Present results of all sensitivity analyses conducted to assess the robustness of the synthesized results.	9, 10, 11
Reporting biases	21	Present assessments of risk of bias due to missing results (arising from reporting biases) for each synthesis assessed.	8
Certainty of evidence	22	Present assessments of certainty (or confidence) in the body of evidence for each outcome assessed.	8
**DISCUSSION**			
Discussion	23a	Provide a general interpretation of the results in the context of other evidence.	12
	23b	Discuss any limitations of the evidence included in the review.	12
	23c	Discuss any limitations of the review processes used.	13
	23d	Discuss implications of the results for practice, policy, and future research.	13
**OTHER INFORMATION**			
Registration	24a	Provide registration information for the review, including register name and registration number, or state that the	6

Section and Topic	Item #	Checklist item	Location where item is reported
and protocol		review was not registered.	
	24b	Indicate where the review protocol can be accessed, or state that a protocol was not prepared.	6
	24c	Describe and explain any amendments to information provided at registration or in the protocol.	6
Support	25	Describe sources of financial or non-financial support for the review, and the role of the funders or sponsors in the review.	16
Competing interests	26	Declare any competing interests of review authors.	16
Availability of data, code and other materials	27	Report which of the following are publicly available and where they can be found: template data collection forms; data extracted from included studies; data used for all analyses; analytic code; any other materials used in the review.	16

Supplementary table 2: Baseline characteristics of included studies.

**Study ID**	**Groups**	**Age (mean,SD)**	**Sex (Male) Number, Percent**	**BMI (mean, SD)**	**Clinical background**													**Clinical Presentation**							
					Hypertension	DM	Smoking	COPD	Hyperlipidemia	Renal insufficiency	Coronary artery disease	Aortic valve regurgitation > 2 degree	Previous cardiac operation	Marfan Syndrome	PVD	CPR (Number, Percent)	Cardiac Tamponade	Aortic Rupture	Pericardial effusion	Neurological Dysfunction	Malperfusion	Depakey type 1	Depakey type 2	Location of the primary tear
**Bojko 2022**	≥80y	(83.5,1.14)	28(40)	(26, 1.92)	63 (90.0)	4 (5.7)	12 (17.1)	10 (14.3)		0 (0.0)	3 (27.3)			0 (0.0)	27 (38.6)	7 (10.0)	13 (18.6)	29 (41.4)		12 (17.1)	11 (15.7)			Tear in the arch
	≥80y	(74.3,2.24)	84(50.9)	(27.6,2.79)	150 (90.9)	34 (20.6)	39 (23.6)	34 (20.6)		4 (9.5)	6 (14.3)			2 (1.2)	63 (38.2)	8 (4.8)	23 (13.9)	46 (27.9)		18 (10.9)	42 (25.5)			
**Nakai 2022**	≥80y	(84.2,4.66)	3(9.4)							1(3.1)	1(3.1)	0			2(6.3)	1(3.1)	15(46.9)			8(25)				The location of the intimal tear was in the ascending aorta or proximal aorticarch (lesser curvature of the transverse arch, opposite the brachiocephalic artery) or unknown.
	≥80y	(75, 4.56)	17(28.8)							3(5.1)	6(10.2)	1(1.7)			4(6.8)	5(8.5)	32(52.4)			13(22)				The location of the intimal tear was in the ascending aorta or proximal aorticarch (lesser curvature of the transverse arch, opposite the brachiocephalic artery) or unknown.
**Dumfarth 2017**	≥80y	(83, 2.5)	33 (43.3)	(26.1 , 5.3)	54 (80.6)	15 (22.4)		8 (11.9)	24 (37.3)		18 (26.9)		8 (11.9)		9 (13.4)	5(7.6)	20 (29.9)	4 (40)	24 (35.8)	6 (9.1)	11 (16.7)	36 (53.7)	31 (46.3)	Tear or ulceration located in the convexity of the aortic arch,which could not be excluded by partial arch replacement
**Hata 2008**	≥80y	81.7 (2.3)	15(35.7)							6(24)		7(16.7)					20(47.6)			9 (21.4)	0			They observe a long intimal tear on the midportion of the aortic arch during DHCA, we have to clamp the proximal arch again,
**Hata2010**	≥80y	81.7 (2.2)	8(29.6)							1(3.7)						1(3.7)	15(55.6)							The intimal tear was located on the ascending aorta to the proximal arch in 20 patients (74.1%). In 7 patients, the intimal tear was found on the distal arch to the descending aorta.
**Kondoh 2016**	≥ 80y	83(1.5)	18 (23)	20.8 (3.15)						5 (6.5)	6 (7.8)	7 (9.1)	1 (1.3)	0		11(12.2)	28 (36.4)			8 (10.4)	2 (2.6)	60 (78)	17(22)	The location of entry in these patients was the ascending aorta and distal arch
**Ahmed 2015**	≥ 80y	82 (2)	29 (74)		28 (72)			21 (54)		14 (36)	15 (38)	12 (31)	2 (5)			2 (5)				4(10)				
**Neri 2001**	≥ 80y	83 ± 3.2	17(70)		24 (100%)	7 (29%)	16 (66%)	17 (70%)		7 (29%)	10 (41)		4 (16%)	0	16 (66%)	0	15 (62%)	7 (29%)	22 (91%)	10 (41%)	Myocardial ischemia 8 (33%) Cerebral ischemia 10 (41%)			Ascending aorta 11 (45%) Transverse arch 13 (54%)

																					Visceral ischemia 7 (29%) Lower extremities ischemia 7 (29%)			
	< 80y	54 ± 8.5 (	113(65)		154 (89%)	31 (18%)	58 (33%)	81 (47%)		47 (27%)	57 (33)		23 (13%)	19 (11%)	52 (30%)	38 (22%)	76 (44%	28 (16%)	154 (89%)	42 (24%)	Myocardial ischemia 30 (17%) Cerebral ischemia 42 (24%) Visceral ischemia 31 (18%) Lower extremities ischemia 28 (16%)			Ascending aorta 151 (87%) Transverse arch 22 (13%)
**Piccardo 2009**		82 (2)	30 (52)		43 (75)	0	11 (19)	5 (8.7)			7 (12)			0		2 (3.5)				11 (19.3)	Mesenteric ischemia 3 (5.2) Shock 13 (22.8)			
**Shiono 2006**	≥ 80y	82(2.4)	10(41.3)		23 (95.8%)		8 (33.3%)				4 (16.7%)			0		2 (8.3)	11 (45.8%)			8(33.3%)	Limb ischemia 1 (4.2%) Visceral ischemia 1 (4.2%)	16 (66.7%)	7 (29.2%)	ascending aorta or the proximal aortic arch 23(96%)
	< 80y	61.9 (12.6)	52(47.3)		96 (87.2%)		53 (48.2%)				8 (7.2%)			8 (7.3%)		2 (1.8)	46 (41.8%)			28(25.5%)	Limb ischemia 2 (1.8%) Visceral ischemia 0	79 (71.8%)	25 (22.7%)	
**Tang 2013**	≥ 80y	85 3	4(19)		19 (90%)	2 (10%)	2 (10%)	2 (10%)		1 (5%)	6 (29%)			0	10 (48%)		6 (29%)	10(48%)		5(24%)	1 (9%)			
	< 80y	60 12	59(73)		60 (75%)	10 (13%)	17 (21%)	4 (5%)		0	13 (16%)			1 (1%)	41 (51%)		15 (19%)	22(28)		5(7%)	32 (40%)			
**Vanhuyse 2012**			5(33.3)	21.6 ± 2.2	13 (86.7%)			3 (20%)	4 (26.7%)	2 (13.3%)		4 (26.6%)					5 (33.3%)		11 (73.3%)	4 (26.7%)	Limb ischemia 1 (6.6%)			

**Hsu 2020**	≥ 80y	83.7 (2.7)	89 (43.2)		141 (68.4)	39 (18.9)		21 (10.2)		13 (6.3)			4 (1.9)	0 (0.0)	7 (3.4)					30 (14.6)				
	< 80y	57.5 (12.5)	2262 (70.3)		2214 (68.8)	325 (10.1)		112 (3.5)		166 (5.2)			48 (1.5)	84 (2.6)	116 (3.6)					240 (7.5)				
**Kawahito 2018**	≥ 80y	83.1 (2.7)	23(20.5)	0		7 (6.3)	14 (12.5)	3 (2.7)		0	9 (8.0)	19 (17.0)	7(.7)	0		5 (4.5)	39 (34.8)	0		26 (23.2)	21 (18.8)		37 (33.0)	The location of the primary tear was frequently observed in the ascending aorta in the octogenarian group, and DeBakey type II dissection was also more frequent.
	< 80y	62.0 (11.4)	511(55.9)	69 (7.5)		59 (6.5)	315 (34.5)	23 (2.5)		20 (2.2)	29 (3.2)	126 (13.8)	1 (1.3)	28 (3.1)		45 (4.9)	197 (21.6)	13(9.8)		130 (14.2)	292 (31.9)		81 (8.9)	The location of the primary tear was frequently observed in the ascending aorta in the octogenarian group, and DeBakey type II dissection was also more frequent.
**Ohnuma 2016**	≥ 80y	83.7 (3.0)	2635(50.9)		502(54.7)	95(10.3)	275 (30)	33(3.5)		36(3.9)	19(2.1)		12(0.3)	0										
	< 80y	83.7 (3.0)	1081(25.4)		2657 (62.4)	409 (9.6)	2227(52.3)	132 (3.5)		136(3.2)	153 (3.6)		1(0.9)	42(1)										
**Omura 2017**	≥ 80y	83.7 (3.0)	28 (44.4)							3 (4.8)	2 (3.2)	8 (12.7)			3 (4.8)					6 (9.5)	12 (19.0)			
	< 80y	65.1 (10.7)	150 (53.2)							19 (6.7)	11 (3.9)	33 (11.7)			24 (8.5)					34 (12.1)	67 (23.8)			
**Suenaga 2016**	≥ 80y	83.9 ± 2.8	6 (24 %)		19 (76 %)														21 (84 %)		6 (24 %)	9 (36 %)		
	< 80y	68.1 ± 9.1	18 (33 %)		35 (64 %)														28 (51 %)		16 (29 %)	31 (56 %)		
**Tochii 2016**	≥ 80y	83± 3	5(20.8)										0	0						2 (8.3)	0		9 (37.5)	
	< 80y	62± 13	74(55.2)										3 (2.2)	5 (3.7)						16 (11.9)	13 (9.7)		27 (20.1)	
**Suzuki 2019**	≥ 80y	84.5 (4.2)	14 (25.5)	21.4 (3.4)	33 (60)	6 (10.9)	11 (20)	5 (9.1)	14 (25.5)	5 (9.1)	8 (14.5)			0	1 (1.8)	5 (9.1)	19 (34.5)			Old cerebrovascular accident 4 (7.3)<br>Cerebral ischemia 8 (14.5)	9 (16.4)			Ascending aorta 40 (72.7)<br>Aortic arch 9 (16.4)<br>Descending aorta 6 (10.9)
	< 80y	63.7 (12.3)	141 (53.4)	23.8 (3.9)	167 (63.3)	23 (8.7)	122 (46.2)	17 (6.4)	65 (24.6)	17 (6.4)	34 (12.9)			15 (5.7)	12 (4.5)	17 (6.4)	72 (27.3)			Old cerebrovascular accident 20 (7.6)<br>Cerebral ischemia 39 (14.8)	73 (27.7)			Ascending aorta 150 (56.8)<br>Aortic arch 48 (18.2)<br>Descending aorta 66 (25)
**Rylski 2011**	Octogenarian	82.8 (2.3)	26 (31 %)		47 (56.6 %)									0		2 (2.4 %)	26 (31.3 %)			8(9.6%)				

	Septuagenarian	73.7 (2.8)	184 (48%)		219 (57.5%)									4 (1.1%)		30 (7.9%)	89 (23.4%)			34(8.9%)				

Supplementary table 2: Baseline characteristics of included studies. (continued)

**Study ID**	**Groups**	**Main procedure**																																**Concomitant procedure**								
		**Ascending**			**Hemi-Arch**			**Total arch replacement**			**Aortic root replacement**			**Bentall**			**Carbol**			**Aorto-Aortic graft**			**David**			**Others**			**Aortic valve valvuloplasty / Replacement**			**CABG**			**Others(name it)**		
**Bojko 2022**	≥ 80y							1	1.4	70	64	91.4	70	6	8.6	70																					
	Septuagenarians							14	8.5	165	132	80	165	33	20	165																					
**Nakai 2022**	≥ 80y	20	62.5	32				1	3.1	32																						0	0	32			
	Septuagenarians	25	42.4	59				10	17	59																						4	6.8	59			
**Dumfarth 2017**	≥ 80y	52	81.3	64				8	0.125	64	6	9.4	64																6	9.4	64	10	15.6	64			
**Hata 2008**	≥ 80y	32	54.7	42	18	42.9	42	1	2.38	42																											
**Hata2010**	≥ 80y	17	63	27	10	37	27																														
**Kondoh 2016**	≥ 80y	73	94.8	77				4	5.2	77	0	0	77							3	3.9	77							1	1.3	77	1	1.3	77			
**Ahmed 2015**	≥ 80y				32	82	39	7	18	39				8	21	39																2	5	39			
**Neri 2001**	≥ 80y	10	42	24	11	46	24	3	12	24				6	25	24	2	8	24	9	37	24							7	29	24	2	8	24	Mitral valve surgery 0	0	24
	< 80y	40	23	173	96	55	173	37	22	173				37	21	173	28	16	173	35	20	173							73	42	173	17	10	173	Mitral valve surgery 9<br>Abdominal aortic fenestration 3	52	173
**Piccardo 2009**		16	28.1	57	27	47.4	57	6	10.5	57	5	8.8	57													3	5.7	57				6	10.5	57			
**Shiono 2006**	≥ 80y					95.8	24	1	4.2	24	0	0	24																0	0	24	1	4.2	24			
	< 80y				82	74.5	110	28	25.5	110	9	8.2	110																2	1.8	110	8	7.3	110			
**Tang 2013**	≥ 80y					85	21	0	0	21				1	5	21							0	0	21	2	10	21				4	19	21			
	< 80y				53	66	80	2	3	80				21	25	80							2	3	80	2	3	80				11	14	80			
**Vanhuyse 2012**		10	66.6	15							2	13.3	15													2	13.3	15				1	6.6	15			
**Hsu 2020**	≥ 80y	130	63.1	206				67	32.5	206	13	6.3	206	13	6.3	206													23	11.2	206	20	9.7	206			
	< 80y	1914	59.5	3217				945	29.4	3217	347	10.8	3217	347	10.8	3217													285	8.9	3217	315	9.8	3217			

**Kawahito 2018**	≥ 80y	94	83.9	112	11	9.8	112	12	10.7	112	1	0.9	112	1	0.9	112													3	2.7	112	2	1.9	112			
	< 80y	550	60.2	914	140	15.3	914	161	17.6	914	53	5.8	914	53	5.8	914													24	2.6	914	66	7.2	914			
**Ohnuma 2016**	≥ 80y				275	30	917							13	1.4	917							3	0.3	917				78	8.5	917	34	3.7	917			
	< 80y				1886	44.3	4258							111	2.6	4258							21	0.5	4258				366	8.6	4258	196	4.6	4258			
**Omura 2017**	≥ 80y	46	73	63	11	17.5	63	9	14.3	63	0	0	63	0	0	63																4	6.3	63			
	< 80y	158	56	282	72	25.5	282	86	30.4	282	19	6.7	282	19	6.7	282																19	6.7	282			
**Suenaga 2016**	≥ 80y	25	100	25																																	
	< 80y	55	100	55																																	
**Tochii 2016**	≥ 80y	23	95.8	24	0	0	24	1	4.2	24	0	0	24																								
	< 80y	88	65.7	134	10	7.5	134	36	26.9	134	18	13.4	134																								
**Suzuki 2019**	≥ 80y		92.7	55				4	7.3	55	0	0	55																5	9.1	55	3	5.5	55	Lower limb bypass 1	1.8	55
	< 80y	245	92.3	264				19	7.2	264	7	2.7	264																16	6.1	264	24	9.1	264	9	3.4	264
**Rylski 2011**	Octogenarian	69	83.1	83	41	49.4	83	8	9.4	83													1	1.2	83	1	1.2	83	replacement 10<br>reconstruction 13	12.1<br>15.7	83	8	9.4	83			
	Septuagenarian	298	78.2	381	199	52.2	381	54	14.2	381													11	2.9	381	6	1.6	381	replacement 28<br>reconstruction 44	7.4<br>11.6	381	69	18.1	381			

Supplementary table 3: NIH tool for assessing risk of bias. NA; not available.

Study ID	1. Was the research question or objective in this paper clearly stated?	2. Was the study population clearly specified and defined?	3. Was the participation rate of eligible persons at least 50%?	4. Were all the subjects selected or recruited from the same or similar populations (including the same time period)? Were inclusion and exclusion criteria for being in the study prespecified and applied uniformly to all participants?	5. Was a sample size justification, power description, or variance and effect estimates provided?	6. For the analyses in this paper, were the exposure(s) of interest measured prior to the outcome(s) being measured?	7. Was the timeframe sufficient so that one could reasonably expect to see an association between exposure and outcome if it existed?	8. For exposures that can vary in amount or level, did the study examine different levels of the exposure as related to the outcome (e.g., categories of exposure, or exposure measured as continuous variable)?	9. Were the exposure measures (independent variables) clearly defined, valid, reliable, and implemented consistently across all study participants?	10. Was the exposure(s) assessed more than once over time?	11. Were the outcome measures (dependent variables) clearly defined, valid, reliable, and implemented consistently across all study participants?	12. Were the outcome assessors blinded to the exposure status of participants?	13. Was loss to follow-up after baseline 20% or less?	14. Were key potential confounding variables measured and adjusted statistically for their impact on the relationship between exposure(s) and outcome(s)?
Bojko 2022	Yes	Yes	Yes	Yes	No	Yes	Yes	Yes	No	Yes	Yes	NA	Yes	Yes
NAkai 2022	Yes	Yes	Yes	Yes	No	Yes	Yes	Yes	Yes	Yes	Yes	No	Yes	Yes
Dumfarth 2017	Yes	Yes	Yes	Yes	No	Yes	Yes	Yes	Yes	Yes	Yes	No	Yes	Yes
Hata 2008	Yes	Yes	Yes	Yes	No	Yes	NA	Yes	Yes	NA	Yes	NA	Yes	Yes
Hata2010	Yes	Yes	Yes	Yes	NA	Yes	NA	NA	Yes	Yes	Yes	No	Yes	Yes
Kondoh 2016	Yes	Yes	Yes	Yes	Yes	Yes	Yes	Yes	Yes	Yes	Yes	No	Yes	Yes
Ahmed 2015	Yes	Yes	Yes	Yes	No	Yes	Yes	Yes	Yes	NA	Yes	NA	Yes	No
Neri 2001	Yes	Yes	Yes	Yes	No	Yes	Yes	Yes	Yes	NA	Yes	NA	No	Yes
Piccardo 2009	Yes	Yes	Yes	Yes	No	Yes	Yes	Yes	Yes	NA	Yes	NA	Yes	Yes
Shiono 2006	Yes	Yes	Yes	Yes	No	Yes	Yes	Yes	Yes	NA	Yes	NA	Yes	Yes
Tang 2013	Yes	Yes	Yes	Yes	No	Yes	Yes	Yes	Yes	NA	Yes	NA	Yes	No
Vanhuyse 2012	Yes	Yes	Yes	Yes	No	Yes	Yes	Yes	Yes	NA	Yes	NA	Yes	No
Hsu 2020	Yes	No	Yes	Yes	Yes	Yes	Yes	No	No	Yes	Yes	No	No	Yes
Kawahito 2018	Yes	Yes	Yes	Yes	No	Yes	Yes	Yes	Yes	Yes	Yes	NA	Yes	Yes
Ohnuma 2016	Yes	Yes	Yes	Yes	Yes	Yes	No	Yes	Yes	Yes	Yes	No	Yes	Yes
Omura 2017	Yes	Yes	Yes	Yes	No	Yes	Yes	Yes	Yes	Yes	Yes	No	Yes	Yes
SueNAga 2016	Yes	Yes	Yes	Yes	No	Yes	Yes	Yes	Yes	NA	Yes	NA	Yes	No
Tochii 2016	Yes	Yes	Yes	Yes	No	Yes	Yes	Yes	Yes	NA	Yes	NA	Yes	No
Suzuki 2019	Yes	Yes	Yes	Yes	No	Yes	Yes	Yes	Yes	NA	Yes	NA	Yes	Yes
Rylski 2011	Yes	Yes	Yes	Yes	No	Yes	No	Yes	Yes	NA	Yes	NA	No	Yes
Benedetto 2021	Yes	Yes	Yes	Yes	No	Yes	Yes	Yes	Yes	Yes	Yes	NA	No	Yes
Chavaron 2006	Yes	Yes	Yes	Yes	No	Yes	Yes	Yes	Yes	Yes	Yes	NA	No	Yes
Goda 2010	Yes	Yes	Yes	Yes	No	Yes	Yes	Yes	Yes	NA	Yes	NA	No	Yes
Trimarchi 2010	Yes	Yes	Yes	Yes	No	Yes	Yes	Yes	Yes	Yes	Yes	NA	NR	Yes
Chen 2022	Yes	Yes	Yes	Yes	No	Yes	Yes	Yes	Yes	NA	Yes	NA	NR	No
Igarashi 2020	Yes	Yes	Yes	Yes	No	Yes	Yes	Yes	Yes	NA	Yes	NA	Yes	Yes
Shimamura 2018	Yes	Yes	Yes	Yes	No	Yes	Yes	Yes	Yes	NA	Yes	NA	Yes	No
